# InlA Promotes Dissemination of *Listeria monocytogenes* to the Mesenteric Lymph Nodes during Food Borne Infection of Mice

**DOI:** 10.1371/journal.ppat.1003015

**Published:** 2012-11-15

**Authors:** Elsa N. Bou Ghanem, Grant S. Jones, Tanya Myers-Morales, Pooja D. Patil, Achmad N. Hidayatullah, Sarah E. F. D'Orazio

**Affiliations:** Department of Microbiology, Immunology, & Molecular Genetics, University of Kentucky, Lexington, Kentucky, United States of America; University of Michigan Medical School, United States of America

## Abstract

Intestinal *Listeria monocytogenes* infection is not efficient in mice and this has been attributed to a low affinity interaction between the bacterial surface protein InlA and E-cadherin on murine intestinal epithelial cells. Previous studies using either transgenic mice expressing human E-cadherin or mouse-adapted *L. monocytogenes* expressing a modified InlA protein (InlA^m^) with high affinity for murine E-cadherin showed increased efficiency of intragastric infection. However, the large inocula used in these studies disseminated to the spleen and liver rapidly, resulting in a lethal systemic infection that made it difficult to define the natural course of intestinal infection. We describe here a novel mouse model of oral listeriosis that closely mimics all phases of human disease: (1) ingestion of contaminated food, (2) a distinct period of time during which *L. monocytogenes* colonize only the intestines, (3) varying degrees of systemic spread in susceptible vs. resistant mice, and (4) late stage spread to the brain. Using this natural feeding model, we showed that the type of food, the time of day when feeding occurred, and mouse gender each affected susceptibility to *L. monocytogenes* infection. Co-infection studies using *L. monocytogenes* strains that expressed either a high affinity ligand for E-cadherin (InlA^m^), a low affinity ligand (wild type InlA from *Lm* EGDe), or no InlA (Δ*inlA*) showed that InlA was not required to establish intestinal infection in mice. However, expression of InlA^m^ significantly increased bacterial persistence in the underlying lamina propria and greatly enhanced dissemination to the mesenteric lymph nodes. Thus, these studies revealed a previously uncharacterized role for InlA in facilitating systemic spread via the lymphatic system after invasion of the gut mucosa.

## Author Summary

Ingestion of *Listeria monocytogenes*-contaminated food can be life-threatening for immune compromised individuals, can cause severe gastroenteritis in otherwise healthy people, and is also thought to occur frequently with little consequence. The factors that determine susceptibility to this infection are unknown, due to the lack of an appropriate animal model that closely mimics this wide range of human disease. Mice are highly resistant to oral *L. monocytogenes* infection, and the prevailing view has been that a low affinity between the bacterial surface protein InlA and E-cadherin expressed on the gut mucosa was largely responsible for limited invasion of the murine intestines. We used a novel food borne model of listeriosis to show that a mouse-adapted InlA offers little advantage over wild type InlA for initial colonization of the gut, and indeed, even bacteria lacking InlA can establish intestinal infection in mice. Thus, other aspects of the murine gastrointestinal environment appear to be the key to innate resistance against oral transmission. Surprisingly, our study uncovered a novel function for InlA later in the infection, when the bacteria begin to spread systemically. The natural feeding model presented here using susceptible and resistant strains of mice should be very useful for future studies investigating both mechanisms of microbial pathogenesis and host responses to oral infection.

## Introduction

*L. monocytogenes* are facultative intracellular bacteria that cause food borne disease in humans ranging in severity from mild, self-limiting gastroenteritis to life-threatening sepsis and meningoencephalitis [1]–[3]. The factors that determine host resistance to intestinal infection and subsequent systemic spread of *L. monocytogenes* are not well understood, primarily due to the lack of a suitable small animal model. Oral infection of mice, for example, is widely perceived to be inefficient, requiring an inoculum of 10^9^–10^11^ bacteria, and is typically not as reproducible as intravenous (i.v.) infection. The low infectivity of *L. monocytogenes* in the gut has long been attributed to a weak interaction between the bacterial surface protein internalin A (InlA) and E-cadherin, a cell adhesion protein expressed on intestinal epithelial cells. InlA has a high affinity for human, rabbit and guinea pig E-cadherin, but does not interact strongly with E-cadherin in rodents [4].

*L. monocytogenes* can directly invade intestinal epithelial cells *in vitro* using a “zipper mechanism” triggered by the binding of InlA to E-cadherin [5], [6]. Pentecost et al. showed that basolaterally expressed E-cadherin was transiently exposed at the tips of intestinal villi as dying cells were extruded from the epithelium, and that *L. monocytogenes* preferentially bound at the multicellular junctions where this occurred [7]. More recently, Nikitas et al. showed that E-cadherin is also luminally accessible near mucus expelling goblet cells [8]. However, other routes of invasion in the gastrointestinal tract are also possible. Many pathogens are transcytosed across the epithelium by M cells located both in macroscopically visible Peyer's Patches and scattered elsewhere throughout intestinal villi [9], [10]. *L. monocytogenes* were shown to associate with murine M cells both in vivo and in vitro [11]–[15] and internalin B (InlB) was implicated in this process [16]. The bacterial adhesins LAP and Vip have also been implicated in translocation across the gut mucosa [17], [18].

Two approaches have been used to improve the efficiency of oral infection in mice, each focused on modeling the interaction between human E-cadherin and InlA. In one approach, transgenic mice expressing both murine and human E-cadherin were generated. In that study, InlA had the greatest effect on colonization in the cecum and colon of the humanized mice, but importantly, intragastric (i.g.) inoculation of an InlA deletion mutant still resulted in significant colonization of intestinal tissues [19]. As an alternate approach, Wollert et al. generated mouse-adapted *L. monocytogenes* expressing a modified InlA (InlA^m^) that bound mouse E-cadherin with the same affinity as for the wild type InlA::human E-cadherin interaction [20]. Infection with 10^7^ CFU (a dose 100-fold lower than typically used) was possible with this strain; however, in that study, no significant difference in intestinal colonization was observed for the InlA^m^-expressing bacteria compared to wildtype *L. monocytogenes* until 72 hours after i.g. inoculation. Both of these approaches suggested that a high affinity interaction between InlA and E-cadherin was not required to breach the intestinal barrier, and hinted at a possible role for InlA during the later stages of intestinal infection. However, the high degree of variability in bacterial loads after i.g inoculation of *L. monocytogenes* InlA^m^ (up to 1000-fold difference within the same experimental group) made it difficult to distinguish clear phenotypes [20].

The fate of microbes delivered by oral gavage, a process that puts organisms suspended in saline directly into the stomach via a feeding needle, is not well understood, despite its widespread use in models of oral infection [21]. In many reports, i.g administration of *L. monocytogenes* resulted in rapid spread to the spleen and liver within 4–12 hours of inoculation, regardless of the bacterial isolate or mouse strain used [20], [22]–[25]. However, in some studies, no systemic spread was observed until 48 hours post-infection (hpi) [14], [26], [27]. The reason for this variable rate of systemic spread is not known, but could be related to the invasive nature of i.g. inoculation, since minor trauma in the esophagus or stomach could facilitate a mechanism of direct bloodstream invasion. In support of this idea, Kinder et al. recently showed that mice fed from a syringe in the mouth were able to generate oral tolerance against ovalbumin, but mice treated by gavage with a feeding needle were not tolerant and instead generated an ovalbumin-specific systemic antibody response (Heather Bruns, personal communication). Rapid spread to the bloodstream is problematic because *L. monocytogenes* does not need a prolonged period of incubation in the host to begin intracellular replication, and the high inoculum typically used to promote intestinal colonization (10^7^–10^11^ CFU) is several orders of magnitude greater than the systemic lethal dose for mice. It is not currently known how long it takes *L. monocytogenes* to translocate across the intestinal mucosa and spread to peripheral tissues after natural ingestion of contaminated food in either mice or humans.

To clearly delineate the role of InlA during intestinal infection, a better model of oral transmission was needed that relied solely on translocation across the gut mucosa, without the complications that arise from a rapid direct bloodstream invasion. In this paper, we report the development of a food borne model of murine listeriosis that consistently results in a 36–48 h period of infection only in the gastrointestinal tract, followed by varying degrees of systemic spread in susceptible BALB/c/By/J (BALB) versus resistant C57BL/6/J (B6) mice. Using this non-invasive natural feeding model, we showed that InlA was not required for early colonization of the murine intestines. However, the mouse-adapted InlA^m^ did promote bacterial persistence in the underlying lamina propria and enhanced dissemination to both the mesenteric lymph nodes and spleen, but not the liver.

## Results

### Low dose intragastric infection results in rapid systemic spread of *Lm* InlA^m^

**Figure 1:**
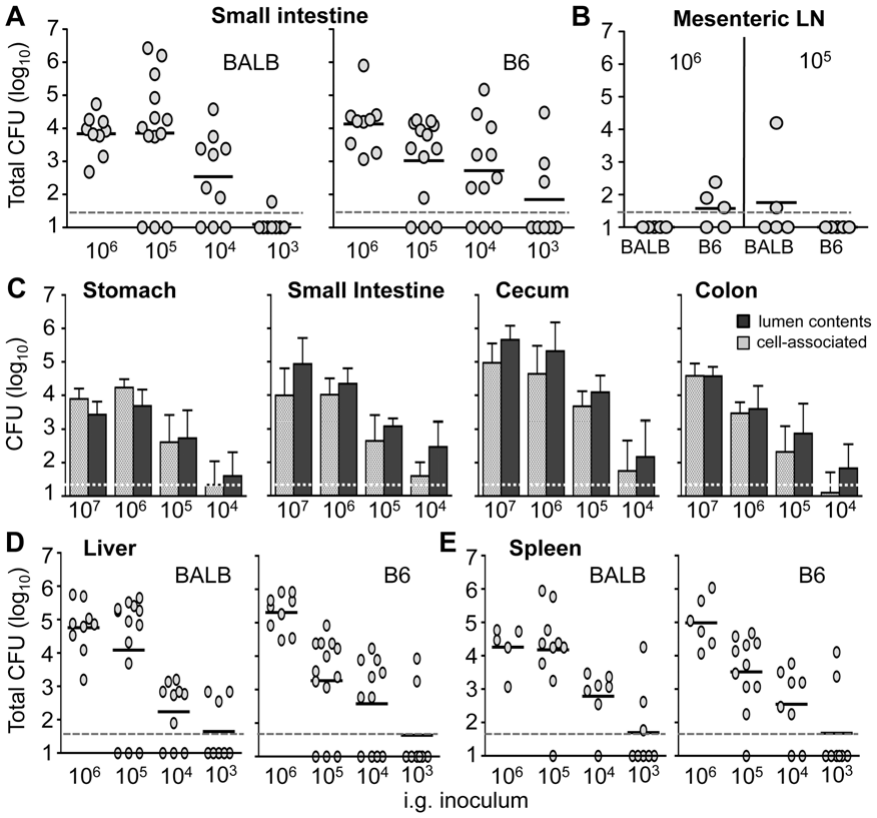
Low dose intragastric infection with mouse-adapted *L. monocytogenes* InlA^m^. Female mice were infected with the indicated inocula by oral gavage and the total number of *L. monocytogenes* CFU (luminal plus cell-associated) in *(A)* the small intestine, *(B)* mesenteric lymph nodes (LN), *(D)* liver, and *(E)* spleen was determined 24 hpi. Symbols represent values for individual mice; horizontal lines indicate the mean for each group. Pooled data from 2–3 separate experiments are shown. *(C)* Mean values +/− SE for *L. monocytogenes* found in either the luminal contents or cell-associated (adherent plus intracellular) in tissue homogenates 24 h after infection of B6 mice (n = 4) are shown. Dashed horizontal lines indicate the limit of detection for each tissue.

The first goal of this study was to develop an improved murine model that could be used to clarify the role of InlA in establishing intestinal infection following oral transmission of *L. monocytogenes*. The most important criterion for the model was a reproducible phase of gastrointestinal infection that preceded systemic spread, to ensure that all bacteria from the initial inoculum that reached the spleen or liver had translocated across the gut mucosa. Our initial strategy was to use i.g. infection at doses lower than previously reported to avoid overwhelming innate resistance mechanisms in the gut and inadvertently facilitating rapid, direct bloodstream invasion by *L. monocytogenes*. Groups of BALB/c/By/J (BALB) and C57BL/6J (B6) mice were infected with 10^3^, 10^4^, 10^5^, or 10^6^ CFU of mouse-adapted *L. monocytogenes* that expressed a modified InlA protein (InlA^m^) [20]. The total number of *L. monocytogenes* present in either the small intestine (Fig. 1A) or the large intestine (not shown) 24 hours post infection (hpi) was proportional to the inoculum given, with a lower limit of approximately 10^4^ CFU for establishing infection in either mouse strain. However, when mice were inoculated with only 10^4^ *Lm* InlA^m^, very few cell-associated bacteria (adherent or intracellular organisms not removed by extensive flushing of the lumen) could be recovered from these tissues (Fig. 1C). Using higher inocula, approximately equal amounts of luminal and cell-associated *L. monocytogenes* were recovered from each tissue.

A dose of at least 10^6^ CFU of *Lm* InlA^m^ was required to achieve consistent intestinal infection in all inoculated mice (Fig. 1A). This inoculum was 10-fold lower than used in the original study published by Wollert et al. [20]. However, i.g. challenge with 10^6^ CFU of *Lm* InlA^m^ resulted in rapid dissemination to both the spleen and liver in all mice tested (Fig. 1D). The route of systemic spread was not likely to have occurred via the lymphatic system since *L. monocytogenes* were found in the draining mesenteric lymph nodes (MLN) of only a few mice (Fig. 1B). Therefore, lowering the challenge dose did not prevent rapid spread of *L. monocytogenes* after i.g. inoculation.

### Infection by ingestion of contaminated food

Since we suspected that i.g. inoculation with a feeding needle was facilitating a direct mechanism of bloodstream invasion that might not be physiologically relevant to human food borne disease, we next set out to develop a less invasive means of orally inoculating mice. To do this, small pieces of *Lm* InlA^m^-contaminated bread were placed in empty cages, and each mouse was allowed to pick up the food and eat it voluntarily. Using this method, at least 10^8^ CFU were required to see cell-associated *L. monocytogenes* in the small intestines of B6 mice (Fig S1A). Importantly, at 24 hpi, there were no bacteria in the spleens or livers of mice fed *L. monocytogenes* (Fig. S1B). Thus, infection by natural feeding resulted in a distinct gastrointestinal phase of infection prior to systemic spread of *L. monocytogenes*. However, intestinal infection was not uniformly observed in all of the mice fed contaminated food, so further optimization of the natural feeding protocol was required.

The composition of gastric secretions varies depending on the type of food ingested, so it was possible that bacterial survival in the gut could vary depending on nature of the contaminated food used for oral transmission. To test this, fecal shedding was monitored in mice fed bread saturated with *Lm* InlA^m^ suspended in either PBS, a glucose solution, or butter. Three hours after ingestion, 50-fold more *L. monocytogenes* had survived passage through the stomach when butter or PBS was used compared with glucose (Fig. S1C). Furthermore, there was a significantly greater number of cell-associated *L. monocytogenes* in the colon 24 hpi, and less variation among mice when butter was used (Fig S1D). Thus, for all subsequent experiments, mice were fed bread saturated with *L. monocytogenes*-contaminated butter.

Mice are typically denied food 16–24 hours prior to i.g. infection to ensure that the stomach is empty enough to receive a 200–500 µl bacterial inoculum. Although this was not necessary for infection by natural feeding, in our pilot studies, mice were fasted to facilitate optimal comparison to the i.g. infection route. To find out if fasting was required for food borne transmission of *L. monocytogenes*, BALB mice were denied food for either 0, 4, or 16 hours. With 0–4 hours of food restriction, only a few of the mice had cell-associated *L. monocytogenes* in the intestines (Fig. S1E). In contrast, when food was denied overnight (16 h), the number of bacteria shed in the feces 3 hpi increased 10 to 100-fold, and importantly, cell-associated *Lm* InlA^m^ were present in the majority of small intestines and in the colons of all mice tested. Thus, in all subsequent experiments, mice were denied food overnight prior to ingestion of *Listeria*-contaminated bread.

### Resistant B6 mice clear ingested *Lm* InlA^m^ faster than susceptible BALB mice

**Figure 2:**
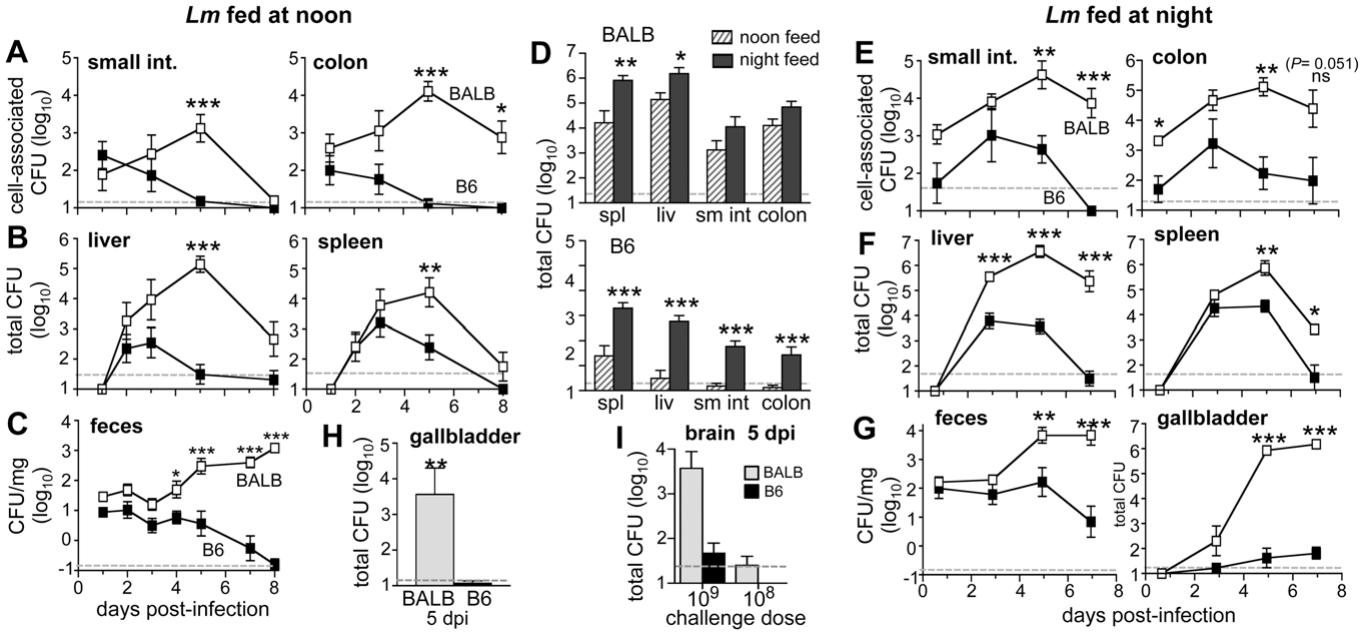
BALB mice are more susceptible than B6 mice to *L. monocytogenes* infection acquired by ingestion of contaminated food. Female BALB (white squares) or B6 (black squares) mice were fed 3–5×10^8^ CFU of *Lm* InlA^m^ either at noon or 9 PM (night) and the number of *L. monocytogenes* present in each tissue was determined over time. In panels *A*, *B*, *E*, *F*, and *G*, groups of mice were sacrificed 1, 3, 5, and 7 dpi. In panels *D, H*, and *I* the indicated organs were harvested 5 dpi. For spleen, liver, small intestines, colon, gall bladder and brain, mean values +/− SD for data pooled from 2 separate experiments (n = 8 mice per group) are shown. Sample groups for fecal analysis in panels *(C)* and *(G)* included 8 mice at all time points for night infection and 22 mice (1 dpi), 15 mice (2 dpi), 14 mice (3 dpi), 12 mice (4 dpi), 9 mice (5 dpi), or 4 mice (7 & 8 dpi) for groups infected at noon. Values for BALB mice that were significantly different from the corresponding B6 group by Mann-Whitney analysis are marked with asterisks. Dashed lines indicate the limit of detection.

Using the optimized parameters, the course of food borne *Lm* InlA^m^ infection was followed in both BALB and B6 mice, strains that are known to have significantly different susceptibility to i.v. challenge with *L. monocytogenes* [28]. Similar loads of cell-associated bacteria were found in the intestines of both mouse strains 24 h after ingestion of *L. monocytogenes* (Fig. 2A). In B6 mice, clearance initiated rapidly, with both cell-associated bacteria and *L. monocytogenes* shed in the feces (Fig. 2C) completely eliminated within 5 to 8 dpi. In contrast, *L. monocytogenes* grew exponentially in the intestines of BALB mice (Fig. 2A). The organisms persisted in the colon, and fecal shedding of *L. monocytogenes* was still detected 8 dpi in BALB mice (Fig. 2C). Thus, resistant B6 mice had a mild, self-limiting gastrointestinal infection while susceptible BALB mice had a more severe infection that persisted in the colon.

By 48 hpi, *L. monocytogenes* had disseminated from the gut to both the spleen and liver (Fig. 2B). The bacterial loads were identical in the spleen and only slightly higher in the livers of BALB mice compared with B6 mice. However, as seen in the intestines, the infection was rapidly cleared in B6 mice, while *L. monocytogenes* continued to replicate exponentially in the organs of BALB mice. The peak of infection occurred 5 days after ingestion of contaminated food, when BALB mice had 100,000-fold more *Lm* InlA^m^ in the liver compared with B6 mice (Fig. 2B). These results suggested that B6 mice had innate resistance mechanisms that could rapidly inhibit the growth of *L. monocytogenes*, and that these mechanisms appeared to be lacking or deficient in BALB mice. Therefore, BALB mice were the preferred strain to test for virulence of *L. monocytogenes* following food borne transmission.

### Time of day influences innate resistance to infection in B6 mice

During the feeding sessions, the B6 mice were generally more receptive to eating contaminated bread pieces than BALB mice. In any given experiment, up to 50% of the BALB mice would not pick up the bread and eat it within 1 hour, while all of the B6 mice ate it within 5–10 minutes. Prior studies showed that BALB mice have a strong food anticipatory rhythm and feed primarily at night [29], [30], but all of our infections had occurred at approximately noon, a time point midway through the 14 hour light cycle for the animals. Reasoning that BALB mice might be more receptive to feeding at night, *L. monocytogenes*-contaminated bread pieces were offered just after the onset of the dark cycle (∼9:30 PM). As expected, both mouse strains readily ate the contaminated food within several minutes during the night feedings.

To find out if night feeding altered the course of *L. monocytogenes* infection, groups of BALB and B6 mice were fed contaminated bread at either noon or 9:30 PM, and bacterial loads were determined 5 dpi. This time point was chosen because it represented the peak of bacterial growth in susceptible BALB mice after noontime feedings, and resistant B6 mice had typically cleared the infection by this point. Night feeding did not significantly alter intestinal infection in BALB mice (Fig. 2D). However, increased bacterial loads were observed in both the spleen and liver of BALB mice infected at night. In contrast, B6 mice were uniformly less resistant to infection in all tissues examined when fed *L. monocytogenes*-contaminated food at night (Fig. 2D). The increased susceptibility of B6 mice was not related to initial colonization rates, as mice infected during the day (Fig. 2A) and mice infected at night (Fig. 2E) both had approximately 10^2^ CFU of *Lm* InlA^m^ in either the small intestine or the colon 24 hpi. The key difference for B6 mice infected at night was that the number of cell-associated *L. monocytogenes* in the gut increased between days 1 and 3 post-infection, prior to the onset of clearance that initiated by 5 dpi (Fig. 2E). This suggested that innate resistance mechanisms in the B6 gut normally capable of inhibiting the rapid exponential growth of *L. monocytogenes* were either delayed or not activated when the food borne transmission of infection occurred at night.

Growth curves in the spleen were similar whether mice were infected during the day or at night, with the greatest difference between BALB and B6 mice occurring 5 dpi (Fig. 2F). In the liver, the largest difference between mouse stains was delayed until 7 dpi, when *L. monocytogenes* had been cleared from the livers of B6 mice and BALB mice had an average of 2.34×10^5^ CFU per liver (Fig. 2F). Prolonged growth in the spleen and liver is thought to lead to a secondary wave of bacteremia and further systemic spread of *L. monocytogenes* to tissues such as the brain [31]. We were unable to detect *L. monocytogenes* in the brains of most mice fed 10^8^ CFU. However, infection with 10^9^ CFU did result in spread of *L. monocytogenes* to the brain following either noontime (not shown) or night (Fig. 2I) feeding. Preliminary studies indicated that a feeding dose of 5×10^9^ CFU was near the LD_50_ for BALB mice (Fig. S2). We concluded from these results that B6 mice could readily be infected at any time of day, but were most resistant to infection when fed *L. monocytogenes*-contaminated food at approximately midday. For susceptible BALB mice, time of day did not significantly alter the course of infection, but night infection was preferable since the animals were more receptive to feeding during their dark cycle.

### The gall bladder is not a significant reservoir for *L. monocytogenes* growth in B6 mice

Hardy et al. recently showed that extracellular *L. monocytogenes* accumulated in the gall bladders of BALB mice infected by the i.g route, and that the bacteria could be excreted back into the intestines upon subsequent feeding [32], [33]. Using the food borne model of infection, we also found significant numbers of *L. monocytogenes* in the gall bladders of BALB/c/By/J mice (Fig. 2G & 2H). However, we were unable to detect *L. monocytogenes* in the gall bladders of resistant B6 mice that were fed *L. monocytogenes* during the day (Fig. 2H). Following night feeding, a few bacteria were found in the B6 gall bladder beginning at 5 dpi, but the bacterial load did not increase significantly over the next two days (Fig. 2G). In contrast, *L. monocytogenes* increased more than 1000-fold from 3 to 5 dpi in BALB gall bladders. These data suggest that B6 mice have innate resistance mechanisms that can prevent dissemination and possibly extracellular growth of *L. monocytogenes* in the gall bladder. Furthermore, the rapid exponential growth of *L. monocytogenes* in the gall bladders of BALB mice may contribute to the persistence of intestinal infection that we observed following either day or night feeding.

### Female mice are more susceptible to food borne listeriosis than male mice

**Figure 3:**
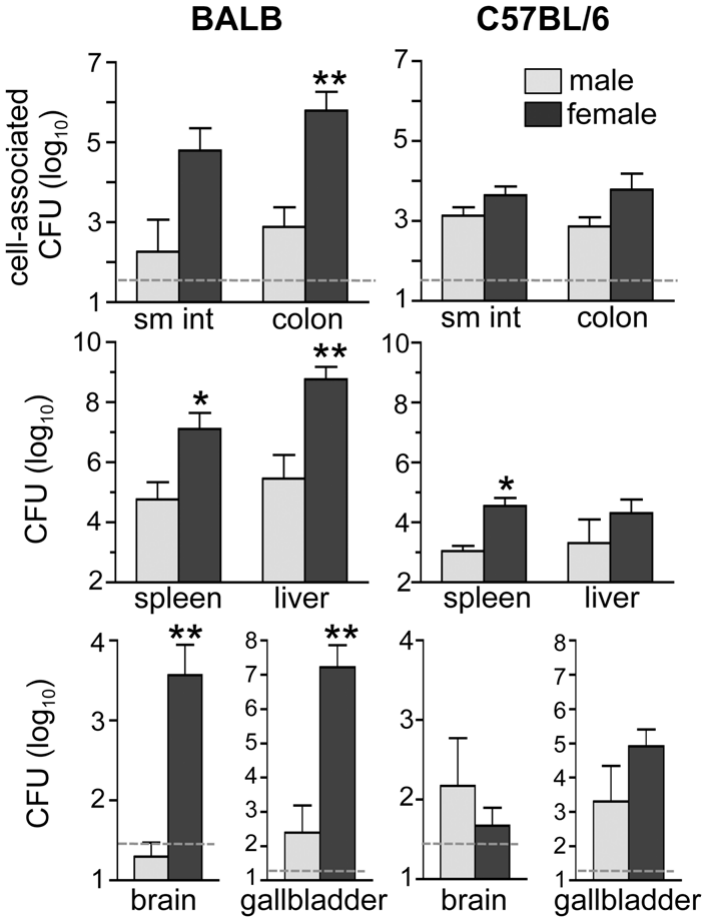
Female BALB, but not B6 mice, are more susceptible than males to food borne listeriosis. Groups of mice (n = 4) were fed 5×10^9^ *Lm* InlA^m^ at night and bacterial loads were determined 4.5 dpi. Mean values +/− SD from one of two separate experiments are shown. Dashed lines indicate limits of detection.

Pasche et al. previously showed that female mice were more susceptible to infection than males, as measured by both survival and colony counts after i.v. injection of *L. monocytogenes* [34]. To test whether females were also more susceptible to food borne infection, *Lm* InlA^m^-contaminated bread was fed to groups of BALB and B6 mice and the number of CFU present in various tissues 5 days later was determined. In BALB mice, significantly greater numbers of *L. monocytogenes* were recovered from females, with at least 100-fold higher loads in the gut, spleen, liver, and brain (Fig. 3). The greatest difference was observed in the gallbladder, with at least 10,000-fold more *L. monocytogenes* found in female tissues compared with males. In B6 mice, slightly higher numbers of *Lm* InlA^m^ were recovered from female tissues; however, the only significant difference occurred in the spleen (Fig. 3). Thus, gender was a key factor influencing the susceptibility of BALB mice, but did not contribute significantly to resistance in B6 mice.

### InlA is not required for primary colonization of the intestines following natural feeding

Having established that female BALB mice fed at night were most prone to infection, we then tested whether expression of the mouse adapted InlA^m^ was required for intestinal colonization in mice following food borne transmission of *L. monocytogenes*. To do this, we assessed the ability of the mouse-adapted strain (expressing the modified InlA^m^) to compete with either wild type *Lm* EGDe (a strain that expressed an InlA protein with a low affinity for murine E-cadherin) or *Lm* Δ*inlA* (a deletion mutant strain that lacked InlA) in mice co-infected with two *L. monocytogenes* strains at a 1∶1 ratio. The number of cell-associated CFU in the gut was determined at both an early (16 h) and later (60 h) time point during the infection. Only the terminal third of the small intestine (approximating the ileum) was examined for these experiments, because a pilot study showed very little colonization of either the duodenum or jejunum using the natural feeding model (Fig. S3).

**Figure 4:**
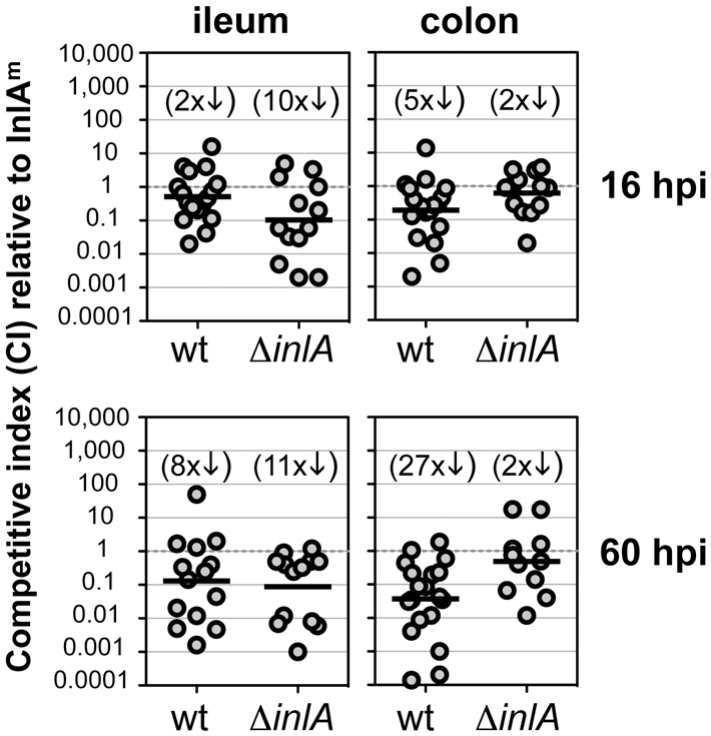
InlA enhances, but is not required for colonization of the murine intestines. Female BALB mice were co-infected at night with a 1∶1 mixture of *Lm* InlA^m^ and either wild type (wt) *Lm* EGDe or *inlA* deletion mutant (Δ*inlA*) for a total inoculum of 7–9×10^8^ CFU. The total number of each *Listeria* strain found in either the ileum or colon was determined at both 16 and 60 hpi. Only the terminal third of the small intestine (approximating the ileum) was harvested because a pilot study showed that the majority of *L. monocytogenes* colonization occurred in the distal portion of the small intestine (Fig. S3). Pooled data from three separate experiments are plotted as competitive indices (CI) to show the ratio of either wt/InlA^m^ or Δ*inlA*/InlA^m^ recovered from each individual mouse. The geometric mean for each group was compared to the theoretical value of 1.0 and the fold difference is shown in parentheses above.

Since InlA is proposed to enhance the efficiency of intestinal infection by promoting rapid invasion of enterocytes and goblet cells [8], [24], we predicted that significantly more *Lm* InlA^m^ would be recovered at the early time point. Instead, we found that the mouse-adapted *L. monocytogenes* strain had only a slight advantage in colonization of the intestines at 16 hpi. In the colon, only 5-fold fewer wild type *Lm* EGDe were recovered compared to *Lm* InlA^m^ and the *inlA* deletion mutant had just a 2-fold defect (Fig. 4). The greatest difference was observed in the ileum, where on average, the mouse adapted InlA^m^ strain outcompeted the deletion mutant by 10-fold. It is unlikely that co-infection with InlA^m^-expressing bacteria promoted invasion of the other strains because similar bacterial titers were observed when mice were infected singly with only *Lm* Δ*inlA* or wt EGDe (data not shown). These results indicated that *L. monocytogenes* lacking a high affinity ligand for E-cadherin could readily establish intestinal infection following ingestion of contaminated food. Therefore, the mouse adapted InlA^m^ protein was not an essential factor needed for food borne transmission of *L. monocytogenes*.

By 60 hpi, the colonization defect for wildtype *Lm* EGDe had increased four- to five-fold in both the ileum and the colon (Fig. 4). The greatest difference was observed in the colon, where the InlA^m^ strain outcompeted the wild type by 27-fold. Surprisingly, the colonization defect for the *inlA* deletion strain did not change significantly from 16 to 60 hpi (Fig. 4). These data suggested that the mouse-adapted *L. monocytogenes* strain did not have an intrinsic growth advantage, but rather that bacteria expressing the wild type InlA were impaired for either growth or persistence in the intestines.

### Wild type InlA impairs bacterial dissemination to the murine MLN and spleen

**Figure 5:**
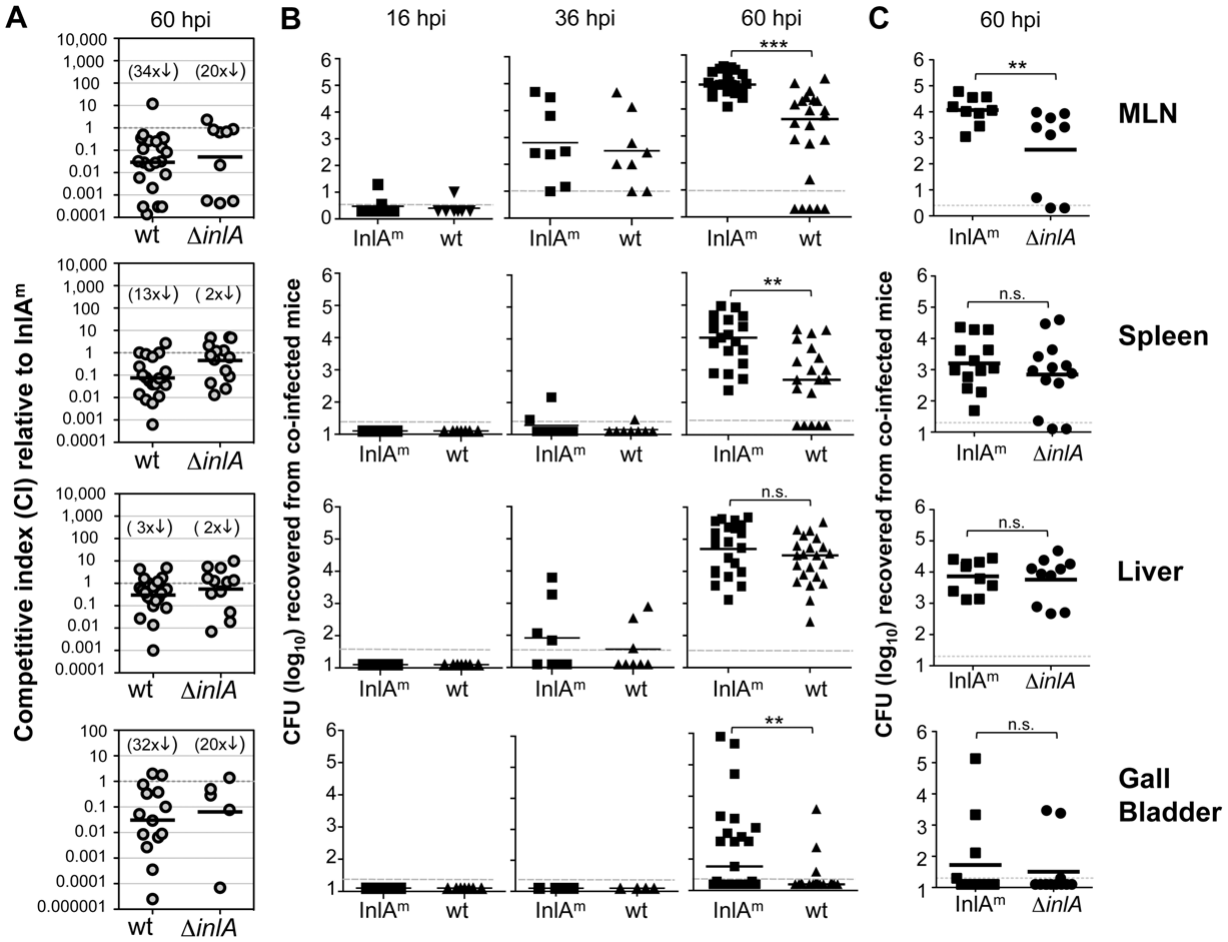
InlA^m^ enhances spread to the mesenteric lymph nodes and spleen. Female BALB mice were co-infected at night with a 1∶1 mixture of *Lm* InlA^m^ and either wild type (wt) *Lm* EGDe or an *inlA* deletion mutant (Δ*inlA*) for a total inoculum of 5–7×10^8^ CFU and the total number of each *Listeria* strain found in the mesenteric lymph nodes (MLN), spleen, liver, and gall bladder was determined. Pooled data from at least two separate experiments are shown. In panel A, the data are plotted as competitive indices (CI) to show the ratio of either wt/InlA^m^ or Δ*inlA*/InlA^m^ recovered from each individual mouse at 60 hpi. The geometric mean for each group was compared to the theoretical value of 1.0 and the fold difference is shown in parentheses above. The actual number of CFU recovered in each mouse after wt (triangles) plus InlA^m^ (squares) co-infection or Δ*inlA* (circles) plus InlA^m^ are shown in panels B & C, respectively. Horizontal bars indicate mean values for each group; statistical significance was assessed by student's t test. The limit of detection in each tissue is marked by a dashed line.

Because the intestinal colonization defect for wild type *L. monocytogenes* increased over time, we hypothesized that expression of the low affinity ligand for E-cadherin impaired the ability of the bacteria to gain access to an intracellular niche that would allow for both replication and dissemination. To find out if *L. monocytogenes* expressing the mouse-adapted InlA^m^ had a competitive advantage for systemic spread from the gut, female BALB mice were co-infected with *Lm* InlA^m^ and *Lm* EGDe and the bacterial loads in the MLN, spleen, liver, and gall bladder were quantified at various time points post-infection. By 36 hpi, small numbers of both *L. monocytogenes* strains had trafficked to the MLN in each mouse tested (Fig. 5B). One day later, at 60 hpi, InlA^m^-expressing *L. monocytogenes* outnumbered wild type *Lm* EGDe by an average of 34-fold (Fig. 5A). A closer examination of the bacterial loads 60 hpi revealed a strikingly consistent number of *Lm* InlA^m^ in the MLN of each mouse tested (Fig. 5A). In contrast, wild type *L. monocytogenes* had a bi-modal distribution. In 6 out of 23 mice tested (26%), little or no *Lm* EGDe was recovered, while the remaining 17 mice had bacterial loads that were 10 to 100-fold lower than *Lm* InlA^m^. In mice co-infected with *Lm* InlA^m^ and the Δ*inlA* mutant, the deletion strain also had a significant colonization defect (Fig. 5A), and showed the same bi-modal distribution (Fig. 5C) in the MLN. These data strongly suggested that the mouse-adapted InlA^m^ promoted, but was not required, for passage through a key bottleneck to exit the intestinal lamina propria and traffic to the draining lymph nodes.

After reaching the MLN, bacteria can transit further via the lymphatic system, eventually draining into the blood, where they are rapidly filtered in either the spleen or liver. In the spleens of the co-infected mice, InlA^m^-expressing *L. monocytogenes* had a 13-fold advantage over wild type *Lm* EGDe (Fig. 5A), and the wild type bacteria had bi-modal distribution similar to that observed in the MLN (Fig. 5B). Wollert *et al*. previously showed that InlA^m^-expressing *L. monocytogenes* had no growth or survival advantage compared to wild type bacteria in the spleen following i.v. inoculation [20], thus, this result suggests that the low affinity InlA expressed by wild type *L. monocytogenes* impaired dissemination from the MLN to the spleen. In contrast, the *inlA* deletion mutant showed no significant colonization defect in the spleen compared to InlA^m^ expressing bacteria (Fig. 5A, C).

InlA had little impact on spread to the liver. The competitive indexes for co-infected mice showed only a two-fold (Δ*inlA*) or three-fold (wt EGDe) colonization defect (Fig. 5A) and there was no significant difference in the actual number of CFU recovered from the liver (Fig. 5B,C). Similar patterns of spread to the MLN, spleen, and liver were also observed in resistant B6 mice (Fig S4C). Routes of dissemination to the gall bladder are not well understood, and extensive colonization of this organ typically does not occur until 5 days after infection of susceptible BALB mice (Fig. 2). However, even at 60 hpi, we found a significantly greater number of InlA^m^ expressing *L. monocytogenes* in the gall bladder compared with wild type *Lm* EGDe (Fig. 5). Together, these results suggested that expression of wildtype InlA impaired systemic spread to tissues of the lymphatic system (MLN and spleen) and the gall bladder, but did not impact efficient dissemination to the liver.

### Intracellular growth of *L. monocytogenes* occurs primarily in the lamina propria of the colon

**Figure 6:**
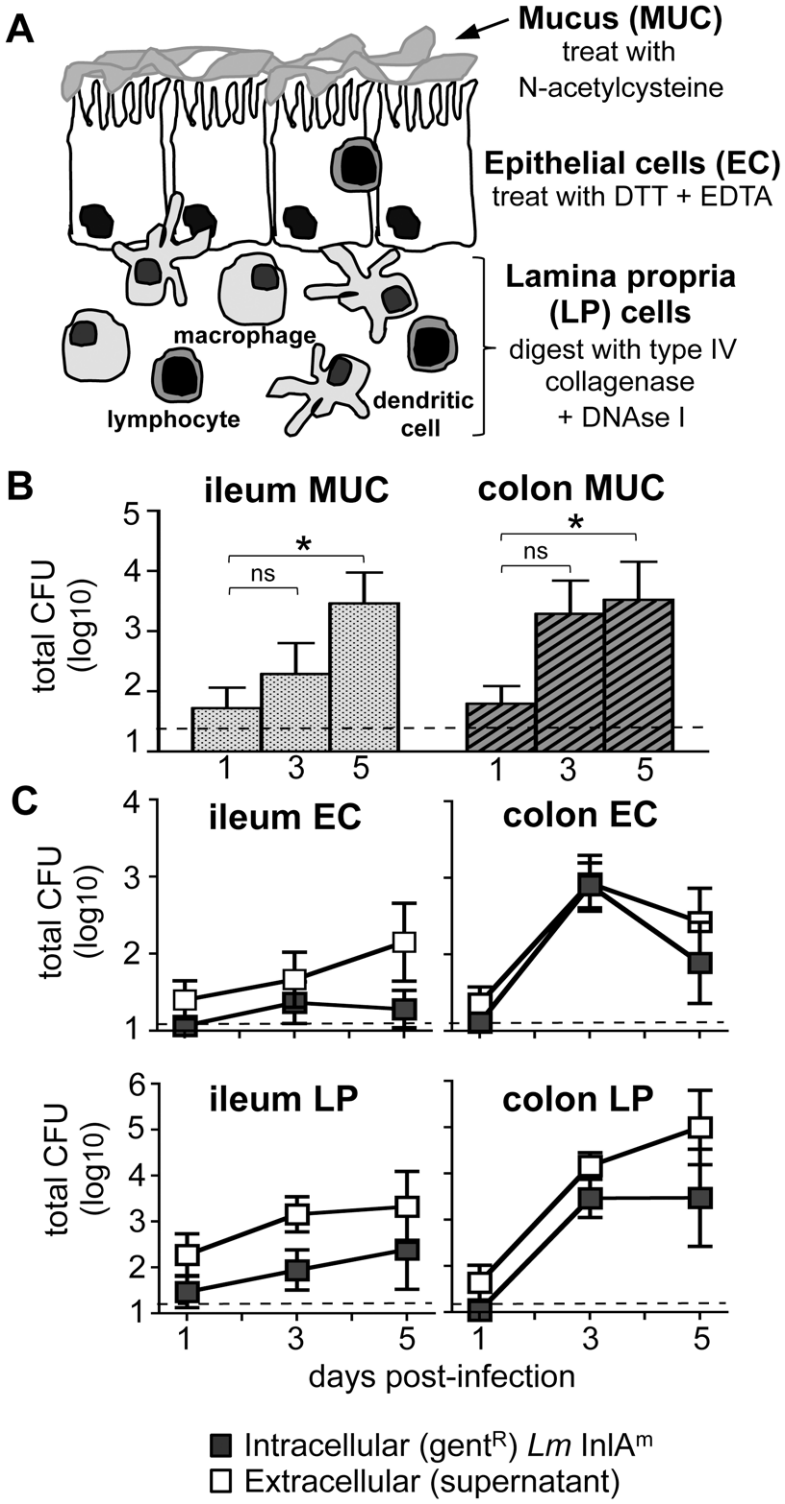
*L. monocytogenes* reside in both extracellular and intracellular compartments in the gut mucosa. *(A)* Intestinal tissues were separated into three fractions: the mucus layer (muc), epithelial cells (EC) and the underlying lamina propria (LP) cells. BALB mice (n = 4 per time point) were fed 2×10^9^ *Lm* InlA^m^ at night. *(B)* Mean values +/− SE for the total number of *Lm* InlA^m^ in the mucus layer and *(C)* the number of intracellular (gentamicin resistant) and extracellular (supernatant fraction) *Lm* InlA^m^ in the LP and EC layers of the ileum and colon are shown. Data from one of two separate experiments are shown.

The striking distribution of *L. monocytogenes* strains in the MLN 60 hpi suggested that the mouse-adapted InlA^m^ protein could enhance the ability of the bacteria to disseminate from the gut to the draining lymph nodes. *L. monocytogenes* could traffic extracellularly in the lymph directly to the MLN, or be carried inside migratory phagocytes such as dendritic cells. This type of “stealth transport” is thought to augment dissemination by protecting the bacteria from mechanisms of immune clearance [35]–[37]. If expression of InlA^m^ helped to promote transit of *L. monocytogenes* inside a migratory phagocyte, then one would expect to find a larger number of InlA^m^-expressing bacteria residing in cells within the lamina propria underlying the intestinal epithelium. To test this, we needed to be able to quantify the total number of bacteria in each compartment of the intestinal tissue. Since microscopic approaches would only identify foci of infection, and not the total number of bacteria, we chose to modify enzymatic digestion methods routinely used for the isolation of intestinal lymphocytes [38], [39] to separate gut tissues into three fractions: the mucus layer, epithelial cells (EC), and lamina propria (LP) cells (Fig. 6A). Single cell suspensions of either EC or LP cells were treated with 25 µg/ml gentamicin and then lysed to define the total number of intracellular *L. monocytogenes* in each cell type. During the processing of each fraction, all washes were collected and centrifuged so the total number of extracellular bacteria present in the supernatant could also be determined.

As shown in Fig. 6B, the number of InlA^m^-expressing *L. monocytogenes* present in the mucus layer of female BALB mice increased over time in both the ileum and the colon. At 24 hours post-infection, very few intracellular InlA^m^ *L. monocytogenes* were detected in any of the gut tissues (Fig. 6C). Two days later, however, CFU counts had increased in each intestinal fraction, with the majority of the bacterial load present in the colon. By 5 days post-infection, the number of intracellular *Lm* InlA^m^ had decreased in the both the ileal and colonic epithelium, while the bacterial load in the lamina propria was maintained (Fig. 6C). Surprisingly, an equal or greater number of extracellular *L. monocytogenes* were found in both tissues at all time points tested. Together, these results suggested that the bulk of *L. monocytogenes* replication occurred in the colon, and that persistence of infection beyond three days was a result of growth or survival in the lamina propria and the mucus layer, but not the epithelium.

### Wild type *L. monocytogenes* have a persistence defect in the colonic lamina propria

**Figure 7:**
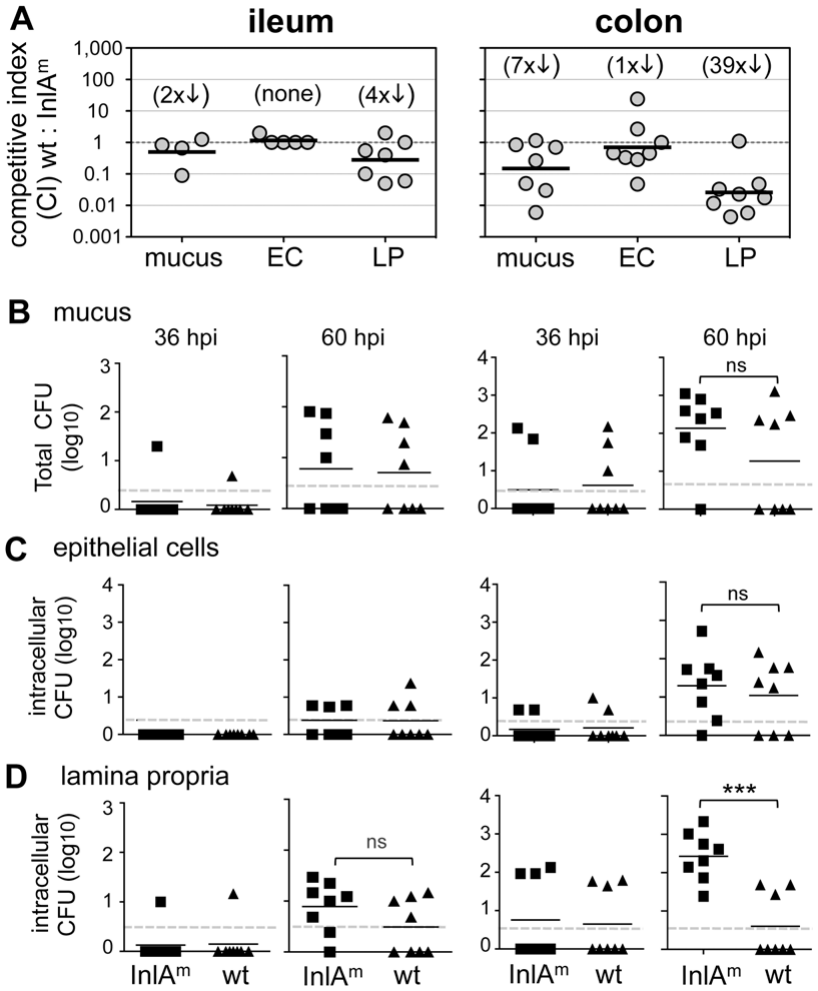
InlA^m^ promotes persistence of *L. monocytogenes* in the lamina propria of the colon. Female BALB mice were co-infected at night with a 1∶1 ratio of InlA^m^ and wild type (wt) *Lm* EGDe for a total inoculum of 7–9×10^8^ CFU. At 36 and 60 hpi, the ileum and colon from each mouse was fractionated, and the total number of each strain found in the mucus *(A, B)* and the number of intracellular (Gent^R^) *Listeria* in either epithelial cells *(A, C)* or lamina propria cells *(A, D)* was determined. Pooled data (n = 8) from two separate experiments are shown. In panel A, the data are plotted as competitive indices (CI) to show the ratio of either wt/InlA^m^ recovered from each mouse 60 hpi. The geometric mean for each group was compared to the theoretical value of 1.0 and the fold difference is shown in parentheses above. The actual number of InlA^m^ CFU (squares) or wt CFU (triangles) recovered from each fraction are shown in panels B, C, and D. Horizontal bars indicate mean values for each group; statistical significance was assessed by student's t test. The limit of detection in each tissue is marked by a dashed line.

Having developed a method that facilitated quantification of the entire bacterial load as *L. monocytogenes* translocated across the gut mucosa, we next asked whether a high affinity interaction between InlA and E-cadherin was needed either for invasion of the epithelium or for persistence in the underlying lamina propria. Female BALB mice were co-infected with a 1∶1 mixture of wild type and InlA^m^-expressing *L. monocytogenes*, and the ileum and colon from each mouse was fractionated and plated at 16, 36 and 60 hpi. At 16 hpi, the total CFU for each strain in mucus and the intracellular CFU in either EC or LP cells was below the limit of detection (data not shown). As expected, very few *Listeria* were detected in the ileum at either 36 or 60 hpi, and there was no significant difference in the number of *Lm* EGDe or *Lm* InlA^m^ recovered (Fig. 7).

In the colon, similar numbers of the two bacterial strains were recovered from the mucus, EC and LP fractions 36 hpi, but the bacterial load remained below the limit of detection in many mice (Fig. 7B, C, D). By 60 hpi, intracellular *L. monocytogenes* were found in the EC fraction of most mice, however, there was not a substantial difference in the number of wild type or InlA^m^-expressing bacteria isolated (Fig. 7A, C). Thus, the mouse-adapted InlA^m^ was not essential for invasion of the colonic epithelium, and expression of the high affinity E-cadherin ligand did not enhance the intracellular replication or survival of *L. monocytogenes* over time in epithelial cells. In contrast, by 60 hpi, InlA^m^-expressing *L. monocytogenes* outcompeted wild type *Lm* EGDe by an average of 39-fold in the colonic lamina propria (Fig. 7A, D). Thus, the mouse- adapted high affinity ligand for E-cadherin promoted either the growth or persistence of *L. monocytogenes* inside cells of the colonic lamina propria.

## Discussion

Oral transmission of *L. monocytogenes* is not highly efficient in mice, and this has been attributed largely to a species specificity for the interaction between the bacterial surface protein InlA and E-cadherin expressed on intestinal epithelial cells. In this study, we developed a novel model of food borne listeriosis in mice and showed that expression of an InlA protein that could serve as a high affinity ligand for E-cadherin was not required for colonization of the murine gut. We propose that the species barrier for InlA is not the major factor responsible for inefficient oral transmission of *L. monocytogenes* in small animal models. Instead, other parameters of the gastric environment are likely to play a much larger role in blocking infection in mice. McConnell et al. showed that the pH of both the stomach and the intestinal tract was lower in mice than in humans [40], and the increased acidity could result in greater bacterial killing. In fact, in this study, very few ingested *L. monocytogenes* survived passage through the murine stomach as evidenced by both CFU counts recovered from the intestinal lumen 24 hpi, and the amount of live *L. monocytogenes* shed in feces 3 hpi. However, prolonged exposure to either the acidic milieu or high osmolarity of the stomach may be essential for *L. monocytogenes* virulence, since these stresses trigger sigma^B^-dependent changes in gene transcription that result in increased invasion of enterocytes and growth in macrophages [41]–[43]. Thus, the small number of *L. monocytogenes* that survive passage through the murine stomach are likely to be better adapted for intestinal colonization. Nonetheless, invasion of the intestinal epithelium appears to be an infrequent event, even when a high affinity interaction between InlA and E-cadherin is possible. Melton-Witt et al. recently estimated that only 1 in 10^6^ *L. monocytogenes* invaded intestinal villi following oral inoculation of guinea pigs [44] and we found a similar frequency of cell-associated bacteria in both the ileum and the colon using a food borne infection model in mice.

*L. monocytogenes* is commonly thought of as an organism that infects the small intestine; however, the colon appeared to be the primary site for bacterial replication in mice following ingestion of contaminated food. In many of the previously published reports of oral listeriosis in mice, the large intestine was not examined. However, our results are consistent with a previous study by Disson et al. that showed increased invasion of *L. monocytogenes* in the colon compared to the small intestine in transgenic mice expressing human E-cadherin [19]. Furthermore, Nikitas et al. recently identified goblet cells as a primary site of intestinal invasion using a ligated jejunal loop model [8], and goblet cells are both more numerous and larger in size in the colon. In that study, the authors used a microscopic approach to show that *L. monocytogenes* lacking InlA were unable to mediate rapid invasion (within 30–45 minutes) of ligated jejunal loops in transgenic mice expressing both murine and human E-cadherin. Intestinal infection was not assessed at later time points, so the ability of luminal bacteria to translocate across the mucosa using other, possibly slower routes, was not determined. Only one other study by Wollert et al. has quantified the amount of InlA^m^-expressing *L. monocytogenes* in the gut at multiple time points following oral transmission, and they also found no difference in the number of wild type or mouse-adapted intracellular bacteria in the small intestine for the first 48 hours after i.g. inoculation [20]. In this study, bacteria completely lacking *inlA* showed a defect only in the ileum, but colonized the colon as efficiently as InlA^m^-expressing *L. monocytogenes*. Although InlA-mediated uptake may be faster, these studies clearly indicate that *L. monocytogenes* can readily use alternate routes, such as passage through M cells, to translocate across the gut mucosa. Furthermore, our data suggest that the invasion mechanisms used by *L. monocytogenes* may differ significantly in the small and large intestines.

The efficiency of enterocyte invasion is not the only factor that determines the net rate of intestinal colonization. Bacterial growth rates, the ability to avoid immune clearance mechanisms, and the rate of dissemination to other tissues, all influence the number of CFU present in the gut at any given time point during infection. However, the route of intestinal invasion may influence the subsequent localization into intestinal compartments with varying degrees of innate resistance against bacterial growth or survival. For example, M cells overlying Peyer's patches deliver phagocytosed bacteria directly to an underlying lymphoid follicle comprised of B cells, T cells, macrophages and dendritic cells. Although the phagocytes in these follicles could provide a replicative niche for *L. monocytogenes*, the close proximity to other immune cells that can rapidly produce IFN-gamma and TNF-alpha may quickly lead to activation of the macrophages, so they no longer support intracellular replication of the bacteria [45], [46]. Resident CD11b(+) CD11c(+) CX3CR1(+) cells in the subepithelial dome of Peyer's patches were recently shown to express significantly higher levels of lysozyme compared with phagocytes found in intestinal villi [47]. *L. monocytogenes* are not killed by lysozyme alone [48], but this observation suggests that there may be subsets of macrophage-like cells in Peyer's patches that have enhanced bactericidal activity and thus, do not support efficient replication of intracellular bacterial pathogens.

In contrast, InlA-mediated uptake occurs primarily at villus tips or near goblet cells and promotes rapid transcytosis of *L. monocytogenes* directly to the underlying lamina propria [7], [8]. Once in the lamina propria, *Listeria* can infect macrophages or dendritic cells, or re-infect epithelial cells by binding to E-cadherin expressed on the basolateral surface. In agreement with Nikitas et al. [8], we found that prolonged infection of intestinal epithelial cells did not occur using the food borne model. This is likely because InlA^m^-expressing bacteria that entered epithelial cells from the apical surface were quickly transcytosed to the lamina propria, and bacteria that infected from the basolateral side were rapidly shed back into the lumen in extruded enterocytes [44]. Interestingly, expression of the low affinity ligand for murine E-cadherin (native InlA) appeared to be deleterious in mice later in the infection, 60 h after ingestion, when wild type *Lm* EGDe was beginning to be cleared from the colon, but *Lm* InlA^m^ persisted. Likewise, Wollert et al. began to observe differences in colonization of the small intestine 72 h after i.g. inoculation of either wildtype or InlA^m^-expressing *L. monocytogenes* [20]. This was not the result of an intrinsic growth or survival advantage for InlA^m^-expressing bacteria, because a deletion mutant lacking *inlA* persisted in the colon equally as well as the mouse-adapted strain. One explanation for these results could be that InlA with a low affinity for E-cadherin may act as a decoy receptor that causes the bacteria to engage non-productively with E-cadherin on the basolateral surface of the epithelium. If the bacteria do not find an intracellular niche in either enterocytes or phagocytes in the lamina propria they would be vulnerable to clearance by innate immune mechanisms.

Although InlA was not required for dissemination to the MLN, InlA^m^-expressing *L. monocytogenes* had a clear advantage in spread from the gut to the draining lymph nodes. In about 20% of the animals we examined, bacteria that lacked a high affinity ligand for E-cadherin (wt EGDE or Δ*inlA*) did not spread to the MLN. This suggests that InlA helps promote passage through a bottleneck in the gut that leads to systemic spread. We presume that this bottleneck is entry into a migratory phagocyte such as a dendritic cell. Although in vitro studies with bone marrow-derived cells suggest that *L. monocytogenes* does not replicate efficiently in dendritic cells, the migratory nature of dendritic cells could serve an important function to promote dissemination of intracellular bacteria via the lymphatic system [49], [50]. In support of this idea, Siddiqui et al. recently identified a minor subset of intestinal dendritic cells that expresses E-cadherin. These monocyte-derived CD103(+)CX3CR1(−) cells accumulated in the intestinal lamina propria during both T cell-mediated colitis and *Trichuris muris* infection, and then migrated to the mesenteric lymph nodes [51], [52]. Indeed, in preliminary studies, we have been able to identify a subset of CD11c(+)E-cadherin(+) cells in the MLN that increased in number during food borne listeriosis (data not shown). InlA^m^-expressing bacteria would thus have an advantage in dissemination, because they would not be limited solely to uptake by phagocytosis and could use InlA-mediated uptake to gain access to an additional subset of migratory phagocytes.

InlA^m^-expressing bacteria had only a slight advantage in reaching the spleen, and no competitive advantage in reaching the liver. Previous studies using tagged strains of either *Yersinia* or *Listeria* in oral inoculation models suggested that there are two possible routes of bacterial dissemination from the gut to the spleen and liver [44], [53]. In the direct pathway, bacteria travel via the portal vein to the liver, Kupffer cells efficiently remove the majority of the bacterial load, and unfiltered organisms continue through the peripheral blood system to reach the spleen. *L. monocytogenes* may access intestinal blood vessels by direct invasion of endothelial cells, a process that is independent of InlA in vitro [54], [55]. A second, indirect pathway occurs when bacteria spread via the lymphatic system, first to the draining lymph nodes, then through efferent lymphatic vessels to the thoracic duct, and then on to peripheral tissues via the bloodstream. Our data differ from the findings of Monk et al. who reported that InlA^m^ promoted spread to both the spleen and the liver [27]. However, that study was performed with mice infected by the i.g. route, so it is possible that physical trauma facilitated direct bloodstream invasion and the large number of bacteria inoculated (1000× more than the i.v. LD_50_) resulted in significant seeding of the spleen as well as the liver.

Another recently described reservoir of *L. monocytogenes* replication in mice is the gall bladder [32]. In this study, we confirmed the observations that *L. monocytogenes* can be recovered from the gall bladders of BALB mice a few days after infection, and that an exponential increase in bacterial load occurred in this tissue. However, very few bacteria reached the gall bladder in B6 mice, and strikingly, there was little increase in the number of *L. monocytogenes* recovered from B6 gallbladders from 3 to 7 dpi. It is possible that the B6 gall bladder is not a permissive site for bacterial replication, or alternatively, continuous spread from other infected tissues such as the spleen or the liver may be a more important factor in determining the overall bacterial load in the gall bladder. The efficiency of gall bladder colonization may also greatly impact the bacterial load in the gut. Upon ingestion of food, the large number of *L. monocytogenes* in the BALB gall bladder could be excreted back into the intestines [33], contributing to the persistent colon infection observed in BALB, but not B6 mice.

During the development of the food borne model of listeriosis, three factors were shown to greatly influence susceptibility to infection: gender, time of day, and food restriction. In susceptible BALB mice, females had the highest bacterial burdens, but the innate resistance of B6 mice did not appear to be gender-dependent. Pasche et al. previously reported increased lethality in female mice infected intravenously with *L. monocytogenes*; however, in that study both BALB and B6 females were more susceptible than males [34]. In another study, B6×C3H F1 mice pre-treated with estrogenic compounds were more susceptible to *L. monocytogenes* [56]. This suggests that estrogen levels in female mice may significantly alter innate resistance to infection. Time of day-dependent changes in immune cell number or function have been reported previously [57]–[59], so it is possible that a circadian rhythm triggered by exposure to light controls the expression of genes needed to rapidly clear *L. monocytogenes*. However, peripheral oscillators that respond to other cues, such as feeding activity, can also establish independent rhythms of gene expression in specific tissues. In that regard, it is notable that a period of food restriction enhanced susceptibility to food borne listeriosis in both B6 (Fig. S1) and BALB (not shown) mice. McConnell et al. showed that the intestines of fasted mice had a higher pH than mice given free access to food [40]. Since decreased acidity would also promote *L. monocytogenes* survival, it is not yet clear exactly how food restriction impacts innate resistance to infection. However, the preliminary data presented here indicate that food borne listeriosis in B6 mice will be a useful model to better understand how circadian rhythms and diurnal variations affect the innate immune system.

The food borne model of *L. monocytogenes* infection has several advantages over the conventional i.g. inoculation model. Transmission of the bacteria occurs by natural feeding, and thus, is not invasive and does not cause unintended minor trauma in the esophagus or stomach. No specialized skills are required for infection, so this method can be widely used by many different laboratories, and may not result in as much lab-to-lab variation as was observed with i.g. inoculation. The model is ideal for studying host response to infection since any mouse strain can be used, including the multitude of knockout and transgenic animals that currently exist, offering an important advantage over use of the recently described guinea pig model [44]. As reported here, susceptible BALB and resistant B6 mice represent the two ends of the spectrum of human disease ranging from mild, self-limiting gastroenteritis to potentially lethal systemic and brain infection. Importantly, it is the first small animal model that can be readily adapted to study the role of particular types of food in transmission of listeriosis. There is a large body of data in the literature examining the growth and survival rates of *Listeria* found in various types of foods, but very little information regarding the infectivity of *Listeria* isolates propagated in different food types or stored at different temperatures [60]–[63]. A large percentage of human listeriosis outbreaks have been associated with foods that are high in fat composition, including one linked to contaminated butter [64]. Ingestion of fatty foods is likely to induce a different profile of gastric secretions that could influence both bacterial survival and the ability to colonize the intestines. Finally, the approach used here should be widely applicable to many other orally transmitted bacterial pathogens such as *Salmonella spp*, *Yersinia enterocolitica* and *Escherichia coli*.

## Materials and Methods

### Ethics statement

This work was performed in accordance with the recommendations in the Guide for the Care and Use of Laboratory Animals published by the National Institutes of Health. All procedures were approved by the Institutional Animal Care and Use Committee (IACUC) at the University of Kentucky.

### Bacteria

Wildtype *L. monocytogenes* EGDe and the modified internalin A derivative (*Lm* InlA^m^) [20] were provided by Wolf Dieter Schubert (Braunschweig, Germany). An *inlA* deletion mutant on the EGDe background (*Lm* Δ*inlA*) was the gift of Cormac Gahan (University College Cork, Ireland). Antibiotic resistant versions of each *L. monocytogenes* were generated for co-infection studies using site-specific integrative plasmids pAD_1_-cGFP and pAD_1_-cYFP [65] (for chloramphenicol resistance; kindly provided by Pascale Cossart, Pasteur, France) or pIMC3 plasmids [66] (for erythromycin, kanamycin and tetracycline resistance; provided by Cormac Gahan). Each strain was intestinally passaged by oral infection of a BALB mouse. Bacteria recovered from the small intestine were grown to early stationary phase in Brain Heart Infusion (BHI) broth shaking at 37°C, and then aliquots were prepared and stored at −80°C. To infect mice, an aliquot was thawed on ice, cultured standing in BHI broth for 1.5 h at 30°C, washed once in PBS, and then suspended in the inoculation solution. Tissue samples were plated on BHI agar (Difco) supplemented with 15 g/L LiCl and 10 g/L glycine (BHI/L+G), a selective medium that inhibited the growth of most intestinal microbiota. Colony growth was monitored after 48 h incubation at 37°C; suspect colonies were confirmed to be *L. monocytogenes* by plating on CHROMagar *Listeria* plates.

### Mice

Male and female C57BL/6/J (B6) and BALB/c/By/J (BALB) mice were purchased from The Jackson Laboratory (Bar Harbor, ME) at 5 weeks of age and used in experiments when they were 6–9 weeks old. All mice were maintained in a specific-pathogen free facility at the University of Kentucky with a 14 h light cycle (7 AM–9 PM) and a 10 h dark cycle (9 PM–7 AM).

### Intragastric infection

Mice were denied food, but given unrestricted access to water 16 h prior to infection. The *Lm* InlA^m^ inoculum was suspended in PBS without bicarbonate and 200 µl was placed directly into the stomachs of non-anesthetized mice [67] using a 20 g straight feeding needle. Food was returned 1 h post-infection.

### Infection by natural feeding

Mice were placed in cages with raised (1 inch) wire flooring (#3 mesh) to prevent coprophagy and food was removed 16–24 h prior to infection unless otherwise indicated. The *L. monocytogenes* inoculum was suspended in 5 µl of either PBS, 2% glucose in PBS, or melted salted butter (Kroger) and used to saturate a 2–3 mm piece of white bread (Kroger) in a microcentrifuge tube. In pilot experiments, blue food coloring was also added to the inoculum to facilitate visual monitoring of the food particle, however, this step was later determined to be unnecessary. At the time of infection, each mouse was placed in an empty cage (no bedding) and the contaminated bread piece was placed on the bottom of the cage. Typically, mice picked up the bread and ate all of it within 5–10 minutes. After eating the bread, mice were returned to their original cages and normal mouse chow was replenished within 30–45 min.

### Cell-associated and luminal *Listeria* in the intestines

Small intestines were either processed whole (Figs. 1–3) or detached and cut into thirds (Figs. 4, 6, 7) approximating the duodenum (proximal), jejunum (middle), and ileum (distal). Colon and cecum sections of the large intestine were processed separately. Intestinal contents were removed by squeezing with sterile forceps, and then each section was flushed with a total of 8–10 ml of PBS through a 25 g needle. To quantify the number of bacteria in the lumen, the pooled contents and flushes were centrifuged for 20 min. at 12,000 *x g*. The bacterial pellet was suspended in 0.5–1.0 ml sterile water and serial dilutions were plated on BHI/L+G agar. Washed intestinal tissues were cut longitudinally with a sterile scalpel blade, placed in 2 ml of sterile water, and then homogenized for 1 minute using a PowerGen 1000 homogenizer (Fisher) at 80% power. The total number of cell-associated (adherent extracellular plus intracellular) bacteria was determined by plating serial dilutions on BHI/L+G agar.

### Tissue homogenates

Spleen, liver and brain were harvested aseptically and homogenized in sterile water for 30 seconds. Gall bladders were collected into microcentrifuge tubes containing 1 ml sterile water, ruptured with sterile scissors, and vortexed for 30 seconds. Mesenteric lymph nodes were mashed through a sterile mesh screen into 0.5 ml of sterile water and each screen was rinsed with an additional 1 ml of water. Dilutions of each tissue sample were prepared in sterile water and plated on BHI/L+G agar. For tissues harvested 16 hours post-infection (hpi), the homogenates were centrifuged for 20 min. at 12,000 *x g* and suspended in 0.1–0.25 ml PBS to lower the limit of detection.

### Fecal analysis

Fecal pellets were collected at the indicated time points, weighed, and then suspended in sterile water (150 mg/ml). Typically, 2–3 pellets with an average total weight of 40 mg were collected from each animal. The pellets were mashed with a sterile toothpick, and then vortexed for 30 seconds before diluting and plating on BHI/L+G agar. Colonies were counted after 24 h growth at 37°C. The limit of detection for *L. monocytogenes* in these samples was 0.13 CFU per mg of feces.

### Co-infections with wild type and InlA^m^ *L. monocytogenes*

For co-infections, bacterial suspensions were mixed prior to saturation of a single bread piece. In early experiments, one strain was tagged with chloramphenicol resistance and tissue homogenates (Fig. 4, 5) or single cell suspensions of intestinal fractions (Fig. 7) were plated on BHI/L+G with or without the presence of chloramphenicol (7 µg/ml). The number of chloramphenicol sensitive (Cm^S^) CFU was determined by subtracting the number of (Cm^R^) colonies from the total CFU found on plates without antibiotic. In later experiments, both of the strains used for co-infection were marked with antibiotic resistance genes and homogenates were differentially plated on BHI with 1 mM IPTG plus 50 µg/ml kanamycin (to detect wildtype *Lm* EGDe), 5 µg/ml erythromycin (to detect *Lm* InlA^m^), or 10 µg/ml tetracycline (to detect *Lm* Δ*inlA)*. Competitive index (CI) ratios were determined by dividing the number of either wild type *Lm* EGDe or Δ*inlA Lm* CFU by the number of *Lm* InlA^m^ CFU recovered from each tissue. If only one strain was recovered, a value equal to the limit of detection was used for the other strain; if no CFU were recovered, then a CI value was not calculated.

### Fractionation of intestinal tissues

Flushed ileum and colon sections were cut longitudinally and treated with N-acetylcysteine (NAC; Sigma) using a variation of a previously described protocol [38] to remove the mucus layer without damaging the underlying epithelium. Each tissue was washed three times by incubating for 2 min. in a tube containing 3 ml of 6 mM NAC, then shaken vigorously before transferring to a fresh tube. The pooled washes were centrifuged for 20 min. at 12,000 *x g*, suspended in sterile water, and vortexed for 30 sec. prior to dilution and plating. No eukaryotic cells (viable or dead) were found in the mucus fractions, as determined by trypan blue staining. The epithelium was removed using standard protocols [39] modified as follows to give higher cell yield and increased cell viability. After mucus extraction, each tissue was cut into small pieces and incubated for 10–20 min. shaking at 37°C in three successive washes (5 ml) of RPMI (Invitrogen # 21870) containing 5% FBS (RP-5), 5 mM EDTA, and 1 mM DTT. The pooled washes (referred to as the EC fraction) were centrifuged at 1,200 *x g* and then the cells and the supernatant were processed separately as described below. The remaining intestinal pieces were rinsed with PBS to remove excess DTT/EDTA, and then digested with collagenase IV (1 mg/ml; Worthington) and DNAse I (40 µg/ml; Worthington) to release the lamina propria (LP) cells. The tissue pieces were incubated for 40 min. shaking at 37°C in 2–3 successive changes of digestion solution (5 ml) until visible pieces of tissue disappeared. The pooled LP fractions were centrifuged at 1,200 *x g* and the cells and supernatant were processed separately.

Supernatants from the EC and LP fractions were centrifuged for 20 min. at 12,000 *x g*, suspended in sterile water, and diluted and plated to determine the total number of extracellular *L. monocytogenes*. The cellularity of single cell suspensions of EC and LP was confirmed by Diff-Quik staining. The cells were incubated for 30 min. at 37°C with 7% CO_2_ in RP-5 containing 25 µg/ml gentamicin to kill any adherent or remaining extracellular *L. monocytogenes*. After gentamicin treatment, the cells were washed twice in PBS, lysed in sterile water, then diluted and plated to determine the number of intracellular *L. monocytogenes*. A comparison of total intracellular plus extracellular CFU recovered from intestinal sections before and after collagenase treatment indicated that the bacteria did not replicate significantly during in vitro processing (not shown).

### Statistics

All statistical analysis was performing using Prism5 for Macintosh (Graph Pad). *P* values less than 0.05 were considered significant and are indicated as follows: *, *P*<0.05; **, *P*<0.01; ***, *P*<0.001; ****, *P*<0.0001.

## Supporting Information

Figure S1 **Optimization of the natural feeding model of** ***L. monocytogenes*** **infection.** *(A)* Total cell-associated CFU in the small intestines of female B6 mice (n = 4) 24 h after ingestion of bread saturated with indicated dose of *Lm* InlA^m^. *(B)* BALB (white circles) and B6 (grey circles) mice (n = 4) were fed 3×10^8^ *Lm* InlA^m^ and the cell-associated (intestines) or total CFU (spleen and liver) was determined 24 and 72 hpi. Bars indicate mean values for each group. *(C, D)* Female B6 mice (n = 6) were fed 3×10^8^ *Lm* InlA^m^ suspended in either glucose, PBS, or melted butter at noon. Mean values +/− SD for *Listeria* shed in the feces *(C)* and the total cell-associated *Listeria* in the small intestine or colon 24 hpi *(D)* are shown. Asterisks indicate mean value significantly different from the mean for the glucose group, as assessed by unpaired t test. *(E, F)* Female BALB mice (n = 7) were denied food for 0 (none), 4 or 16 (O/N) hours and then fed bread pieces saturated with 3–5×10^8^ *Lm* InlA^m^ suspended in butter. The total *Listeria* CFU present in the feces 3 hpi *(E)* and both the luminal and cell-associated *L. monocytogenes* in the small intestines and colon *(F)* was determined 24 hpi. The limit of detection for each organ is indicated by a dashed line.
(TIF)

Figure S2 **The LD_50_ for foodborne transmission in BALB mice is approximately 5×10^9^ CFU.** Female BALB and B6 mice (n = 7) were fed 5×10^9^ *Lm* InlA^m^ at night and survival was monitored over time.
(TIF)

Figure S3 **Food borne transmission of** ***L. monocytogenes*** **results in colonization of primarily the distal third of the small intestine.** Female BALB mice (n = 4) were fed 1×10^9^ CFU of *Lm* InlA^m^ and the total cell-associated CFU in the small intestine was determined over time. Each small intestine was cut into equal thirds approximating the duodenum (proximal), jejunum (medial), and ileum (distal) prior to flushing, homogenization, dilution and plating on BHI/L+G agar. Two-way ANOVA indicated a significant difference in bacterial colonization that varied with the section of the small intestine.
(TIF)

Figure S4 **InlA^m^ enhances systemic spread of** ***L. monocytogenes*** **in B6 mice.** *(A)* Mean values +/−SD for total cell-associated *Listeria* in groups of female B6 mice infected either at noon (day) or 9:30 PM (night) with 4–5×10^8^ CFU of either *Lm* InlA^m^ or *Lm* EGDe are shown. *(B, C, D)* Female B6 mice were co-infected with a total of 8–9×10^8^ CFU of *Lm* InlA^m^ and *Lm* EGDe mixed in a 1∶1 ratio and total number of each strain present in the tissues 16 or 60 hpi was determined. In panel *(D*), ileum and colon were fractionated and the total mucus-associated and intracellular (gent^R^) or extracellular (sup) CFU in both the epithelial cell (EC) and lamina propria (LP) fractions were determined 60 hpi. Bars indicate mean values for each sample group. Dashed lines indicate the limit of detection in each organ.
(TIF)

## References

[1] Bartt R (2000) *Listeria* and atypical presentations of *Listeria* in the central nervous system. Semin Neurol 20: 361–373. 11051300 10.1055/s-2000-9398

[2] Munoz P, Rojas L, Bunsow E, Saez E, Sanchez-Cambronero L, et al (2011) Listeriosis: An emerging public health problem especially among the elderly. J Infect 64: 19–33. 22037557 10.1016/j.jinf.2011.10.006

[3] Ooi ST, Lorber B (2005) Gastroenteritis due to *Listeria monocytogenes* . Clin Infect Dis 40: 1327–1332. 15825036 10.1086/429324

[4] Lecuit M, Dramsi S, Gottardi C, Fedor-Chaiken M, Gumbiner B, et al (1999) A single amino acid in E-cadherin responsible for host specificity towards the human pathogen *Listeria monocytogenes* . The EMBO Journal 18: 3956–3963. 10406800 10.1093/emboj/18.14.3956 PMC1171471

[5] Lecuit M, Ohayon H, Braun L, Mengaud J, Cossart P (1997) Internalin of *Listeria monocytogenes* with an intact leucine-rich repeat region is sufficient to promote internalization. Infect Immun 65: 5309–5319. 9393831 10.1128/iai.65.12.5309-5319.1997 PMC175764

[6] Mengaud J, Ohayon H, Gounon P, Mege RM, Cossart P (1996) E-cadherin is the receptor for internalin, a surface protein required for entry of *L. monocytogenes* into epithelial cells. Cell 84: 923–932. 8601315 10.1016/s0092-8674(00)81070-3

[7] Pentecost M, Otto G, Theriot JA, Amieva MR (2006) *Listeria monocytogenes* invades the epithelial junctions at sites of cell extrusion. PLoS Pathog 2: e3. 16446782 10.1371/journal.ppat.0020003 PMC1354196

[8] Nikitas G, Deschamps C, Disson O, Niault T, Cossart P, et al (2011) Transcytosis of *Listeria monocytogenes* across the intestinal barrier upon specific targeting of goblet cell accessible E-cadherin. J Exp Med 208: 2263–2277. 21967767 10.1084/jem.20110560 PMC3201198

[9] Corr SC, Gahan CC, Hill C (2008) M-cells: origin, morphology and role in mucosal immunity and microbial pathogenesis. FEMS Immunol Med Microbiol 52: 2–12. 18081850 10.1111/j.1574-695X.2007.00359.x

[10] Jang MH, Kweon MN, Iwatani K, Yamamoto M, Terahara K, et al (2004) Intestinal villous M cells: an antigen entry site in the mucosal epithelium. Proc Natl Acad Sci U S A 101: 6110–6115. 15071180 10.1073/pnas.0400969101 PMC395931

[11] Corr S, Hill C, Gahan CG (2006) An in vitro cell-culture model demonstrates internalin- and hemolysin-independent translocation of *Listeria monocytogenes* across M cells. Microb Pathog 41: 241–250. 17049432 10.1016/j.micpath.2006.08.003

[12] Daniels JJ, Autenrieth IB, Goebel W (2000) Interaction of *Listeria monocytogenes* with the intestinal epithelium. FEMS Microbiol Lett 190: 323–328. 11034299 10.1111/j.1574-6968.2000.tb09306.x

[13] Jensen VB, Harty JT, Jones BD (1998) Interactions of the invasive pathogens Salmonella typhimurium, *Listeria monocytogenes*, and Shigella flexneri with M cells and murine Peyer's patches. Infect Immun 66: 3758–3766. 9673259 10.1128/iai.66.8.3758-3766.1998 PMC108412

[14] MacDonald TT, Carter PB (1980) Cell-mediated immunity to intestinal infection. Infect Immun 28: 516–523. 6772561 10.1128/iai.28.2.516-523.1980 PMC550965

[15] Marco AJ, Altimira J, Prats N, Lopez S, Dominguez L, et al (1997) Penetration of *Listeria monocytogenes* in mice infected by the oral route. Microb Pathog 23: 255–263. 9405203 10.1006/mpat.1997.0144

[16] Chiba S, Nagai T, Hayashi T, Baba Y, Nagai S, et al (2011) Listerial invasion protein internalin B promotes entry into ileal Peyer's patches in vivo. Microbiol Immunol 55: 123–129. 21204945 10.1111/j.1348-0421.2010.00292.x

[17] Burkholder KM, Bhunia AK (2010) *Listeria monocytogenes* uses Listeria adhesion protein (LAP) to promote bacterial transepithelial translocation and induces expression of LAP receptor Hsp60. Infect Immun 78: 5062–5073. 20876294 10.1128/IAI.00516-10 PMC2981324

[18] Cabanes D, Sousa S, Cebria A, Lecuit M, Garcia-del Portillo F, et al (2005) Gp96 is a receptor for a novel *Listeria monocytogenes* virulence factor, Vip, a surface protein. EMBO J 24: 2827–2838. 16015374 10.1038/sj.emboj.7600750 PMC1182245

[19] Disson O, Grayo S, Huillet E, Nikitas G, Langa-Vives F, et al (2008) Conjugated action of two species-specific invasion proteins for fetoplacental listeriosis. Nature 455: 1114–1118. 18806773 10.1038/nature07303

[20] Wollert T, Pasche B, Rochon M, Deppenmeier S, van den Heuvel J, et al (2007) Extending the host range of *Listeria monocytogenes* by rational protein design. Cell 129: 891–902. 17540170 10.1016/j.cell.2007.03.049

[21] Boyle JP, Saeij JP, Boothroyd JC (2007) *Toxoplasma gondii*: inconsistent dissemination patterns following oral infection in mice. Exp Parasitol 116: 302–305. 17335814 10.1016/j.exppara.2007.01.010

[22] Czuprynski CJ, Faith NG, Steinberg H (2003) A/J mice are susceptible and C57BL/6 mice are resistant to *Listeria monocytogenes* infection by intragastric inoculation. Infect Immun 71: 682–689. 12540546 10.1128/IAI.71.2.682-689.2003 PMC145353

[23] Gajendran N, Mittrucker HW, Bordasch K, Heinemann E, Koch M, et al (2007) Regional IFNgamma expression is insufficient for efficacious control of food-borne bacterial pathogens at the gut epithelial barrier. Int Immunol 19: 1075–1081. 17698562 10.1093/intimm/dxm075

[24] Lecuit M, Vandormael-Pournin S, Lefort J, Huerre M, Gounon P, et al (2001) A transgenic model for listeriosis: role of internalin in crossing the intestinal barrier. Science 292: 1722–1725. 11387478 10.1126/science.1059852

[25] Pron B, Boumaila C, Jaubert F, Sarnacki S, Monnet JP, et al (1998) Comprehensive study of the intestinal stage of listeriosis in a rat ligated ileal loop system. Infect Immun 66: 747–755. 9453636 10.1128/iai.66.2.747-755.1998 PMC107965

[26] Kursar M, Bonhagen K, Kohler A, Kamradt T, Kaufmann SH, et al (2004) Antigen-specific CD8+ T cell responses in intestinal tissues during murine listeriosis. Microbes Infect 6: 8–16. 14738888 10.1016/j.micinf.2003.10.004

[27] Monk IR, Casey PG, Hill C, Gahan CG (2010) Directed evolution and targeted mutagenesis to murinize *Listeria monocytogenes* internalin A for enhanced infectivity in the murine oral infection model. BMC Microbiol 10: 318. 21144051 10.1186/1471-2180-10-318 PMC3016325

[28] Cheers C, McKenzie IFC, Pavlov H, Waid C, York J (1978) Resistance and susceptibility of mice to bacterial infection: course of listeriosis in resistant or susceptible mice. Infection and Immunity 19: 763–770. 417029 10.1128/iai.19.3.763-770.1978 PMC422254

[29] Holmes MM, Mistlberger RE (2000) Food anticipatory activity and photic entrainment in food-restricted BALB/c mice. Physiol Behav 68: 655–666. 10764895 10.1016/s0031-9384(99)00231-0

[30] Kowal M, Buda-Lewandowska D, Plytycz B, Styrna J (2002) Day/night food consumption in mice is strain and age-dependent. Folia Biol (Krakow) 50: 1–3. 12597524

[31] Berche P (1995) Bacteremia is required for invasion of the murine central nervous system by *Listeria monocytogenes* . Microb Pathog 18: 323–336. 7476097 10.1006/mpat.1995.0029

[32] Hardy J, Francis KP, DeBoer M, Chu P, Gibbs K, et al (2004) Extracellular replication of *Listeria monocytogenes* in the murine gall bladder. Science 303: 851–853. 14764883 10.1126/science.1092712

[33] Hardy J, Margolis JJ, Contag CH (2006) Induced biliary excretion of *Listeria monocytogenes* . Infect Immun 74: 1819–1827. 16495556 10.1128/IAI.74.3.1819-1827.2006 PMC1418634

[34] Pasche B, Kalaydjiev S, Franz TJ, Kremmer E, Gailus-Durner V, et al (2005) Sex-dependent susceptibility to *Listeria monocytogenes* infection is mediated by differential interleukin-10 production. Infect Immun 73: 5952–5960. 16113316 10.1128/IAI.73.9.5952-5960.2005 PMC1231091

[35] Griffin AJ, Li LX, Voedisch S, Pabst O, McSorley SJ (2011) Dissemination of persistent intestinal bacteria via the mesenteric lymph nodes causes typhoid relapse. Infect Immun 79: 1479–1488. 21263018 10.1128/IAI.01033-10 PMC3067558

[36] Vazquez-Torres A, Jones-Carson J, Baumler AJ, Falkow S, Valdivia R, et al (1999) Extraintestinal dissemination of Salmonella by CD18-expressing phagocytes. Nature 401: 804–808. 10548107 10.1038/44593

[37] Voedisch S, Koenecke C, David S, Herbrand H, Forster R, et al (2009) Mesenteric lymph nodes confine dendritic cell-mediated dissemination of *Salmonella enterica* serovar Typhimurium and limit systemic disease in mice. Infect Immun 77: 3170–3180. 19506012 10.1128/IAI.00272-09 PMC2715677

[38] Lange S, Delbro DS, Jennische E (1994) Evans blue permeation of intestinal mucosa in the rat. Scand J Gastroenterol 29: 38–46. 8128176 10.3109/00365529409090435

[39] Lefrancois L, Lycke N (2001) Isolation of mouse small intestinal intraepithelial lymphocytes, Peyer's patch, and lamina propria cells. Curr Protoc Immunol Chapter 3: Unit 3 19. 10.1002/0471142735.im0319s17 18432783

[40] McConnell EL, Basit AW, Murdan S (2008) Measurements of rat and mouse gastrointestinal pH, fluid and lymphoid tissue, and implications for in-vivo experiments. J Pharm Pharmacol 60: 63–70. 18088506 10.1211/jpp.60.1.0008

[41] Conte MP, Petrone G, Di Biase AM, Ammendolia MG, Superti F, et al (2000) Acid tolerance in *Listeria monocytogenes* influences invasiveness of enterocyte-like cells and macrophage-like cells. Microb Pathog 29: 137–144. 10968945 10.1006/mpat.2000.0379

[42] Conte MP, Petrone G, Di Biase AM, Longhi C, Penta M, et al (2002) Effect of acid adaptation on the fate of *Listeria monocytogenes* in THP-1 human macrophages activated by gamma interferon. Infect Immun 70: 4369–4378. 12117947 10.1128/IAI.70.8.4369-4378.2002 PMC128136

[43] Garner MR, Njaa BL, Wiedmann M, Boor KJ (2006) Sigma B contributes to *Listeria monocytogenes* gastrointestinal infection but not to systemic spread in the guinea pig infection model. Infect Immun 74: 876–886. 16428730 10.1128/IAI.74.2.876-886.2006 PMC1360341

[44] Melton-Witt JA, Rafelski SM, Portnoy DA, Bakardjiev AI (2011) Oral Infection with Signature-Tagged *Listeria monocytogenes* Reveals Organ-Specific Growth and Dissemination Routes in Guinea Pigs. Infect Immun 80: 720–732. 22083714 10.1128/IAI.05958-11 PMC3264322

[45] Portnoy DA, Schreiber RD, Connelly P, Tilney LG (1989) Interferon-gamma limits access of *Listeria monocytogenes* to the macrophage cytoplasm. Journal of Experimental Medicine 170: 2141–2146. 2511268 10.1084/jem.170.6.2141 PMC2189551

[46] Thale C, Kiderlen AF (2005) Sources of interferon-gamma (IFN-gamma) in early immune response to *Listeria monocytogenes* . Immunobiology 210: 673–683. 16323704 10.1016/j.imbio.2005.07.003

[47] Lelouard H, Henri S, De Bovis B, Mugnier B, Chollat-Namy A, et al (2010) Pathogenic bacteria and dead cells are internalized by a unique subset of Peyer's patch dendritic cells that express lysozyme. Gastroenterology 138: 173–184 e171–173. 19800337 10.1053/j.gastro.2009.09.051

[48] Rae CS, Geissler A, Adamson PC, Portnoy DA (2011) Mutations of the *Listeria monocytogenes* peptidoglycan N-deacetylase and O-acetylase result in enhanced lysozyme sensitivity, bacteriolysis, and hyperinduction of innate immune pathways. Infect Immun 79: 3596–3606. 21768286 10.1128/IAI.00077-11 PMC3165460

[49] Pron B, Boumaila C, Jaubert F, Berche P, Milon G, et al (2001) Dendritic cells are early cellular targets of *Listeria monocytogenes* after intestinal delivery and are involved in bacterial spread in the host. Cell Microbiol 3: 331–340. 11298655 10.1046/j.1462-5822.2001.00120.x

[50] Westcott MM, Henry CJ, Cook AS, Grant KW, Hiltbold EM (2007) Differential susceptibility of bone marrow-derived dendritic cells and macrophages to productive infection with *Listeria monocytogenes* . Cell Microbiol 9: 1397–1411. 17250592 10.1111/j.1462-5822.2006.00880.x

[51] Mullaly SC, Burrows K, Antignano F, Zaph C (2011) Assessing the role of CD103 in immunity to an intestinal helminth parasite. PLoS One 6: e19580. 21573179 10.1371/journal.pone.0019580 PMC3088701

[52] Siddiqui KR, Laffont S, Powrie F (2010) E-cadherin marks a subset of inflammatory dendritic cells that promote T cell-mediated colitis. Immunity 32: 557–567. 20399121 10.1016/j.immuni.2010.03.017 PMC2938478

[53] Barnes PD, Bergman MA, Mecsas J, Isberg RR (2006) Yersinia pseudotuberculosis disseminates directly from a replicating bacterial pool in the intestine. J Exp Med 203: 1591–1601. 16754724 10.1084/jem.20060905 PMC2118325

[54] Greiffenberg L, Sokolovic Z, Schnittler HJ, Spory A, Bockmann R, et al (1997) *Listeria monocytogenes*-infected human umbilical vein endothelial cells: internalin-independent invasion, intracellular growth, movement, and host cell responses. FEMS Microbiol Lett 157: 163–170. 9418251 10.1111/j.1574-6968.1997.tb12768.x

[55] Parida SK, Domann E, Rohde M, Muller S, Darji A, et al (1998) Internalin B is essential for adhesion and mediates the invasion of *Listeria monocytogenes* into human endothelial cells. Mol Microbiol 28: 81–93. 9593298 10.1046/j.1365-2958.1998.00776.x

[56] Pung OJ, Luster MI, Hayes HT, Rader J (1984) Influence of steroidal and nonsteroidal sex hormones on host resistance in mice: increased susceptibility to *Listeria monocytogenes* after exposure to estrogenic hormones. Infect Immun 46: 301–307. 6500690 10.1128/iai.46.2.301-307.1984 PMC261530

[57] Abo T, Kawate T, Itoh K, Kumagai K (1981) Studies on the bioperiodicity of the immune response. I. Circadian rhythms of human T, B, and K cell traffic in the peripheral blood. J Immunol 126: 1360–1363. 6970770

[58] Fortier EE, Rooney J, Dardente H, Hardy MP, Labrecque N, et al (2011) Circadian variation of the response of T cells to antigen. J Immunol 187: 6291–6300. 22075697 10.4049/jimmunol.1004030

[59] Keller M, Mazuch J, Abraham U, Eom GD, Herzog ED, et al (2009) A circadian clock in macrophages controls inflammatory immune responses. Proc Natl Acad Sci U S A 106: 21407–21412. 19955445 10.1073/pnas.0906361106 PMC2795539

[60] Pearson LJ, Marth EH (1990) *Listeria monocytogenes*–threat to a safe food supply: a review. J Dairy Sci 73: 912–928. 2111832 10.3168/jds.S0022-0302(90)78748-6

[61] Rorvik LM (2000) *Listeria monocytogenes* in the smoked salmon industry. Int J Food Microbiol 62: 183–190. 11156261 10.1016/s0168-1605(00)00334-2

[62] Ryan S, Begley M, Hill C, Gahan CG (2010) A five-gene stress survival islet (SSI-1) that contributes to the growth of *Listeria monocytogenes* in suboptimal conditions. J Appl Microbiol 109: 984–995. 20408910 10.1111/j.1365-2672.2010.04726.x

[63] Shrestha S, Grieder JA, McMahon DJ, Nummer BA (2011) Survival of *Listeria monocytogenes* introduced as a post-aging contaminant during storage of low-salt Cheddar cheese at 4, 10, and 21 degrees C. J Dairy Sci 94: 4329–4335. 21854905 10.3168/jds.2011-4219

[64] Maijala R, Lyytikainen O, Autio T, Aalto T, Haavisto L, et al (2001) Exposure of *Listeria monocytogenes* within an epidemic caused by butter in Finland. Int J Food Microbiol 70: 97–109. 11759767 10.1016/s0168-1605(01)00532-3

[65] Balestrino D, Hamon MA, Dortet L, Nahori MA, Pizarro-Cerda J, et al (2010) Single-cell techniques using chromosomally tagged fluorescent bacteria to study *Listeria monocytogenes* infection processes. Appl Environ Microbiol 76: 3625–3636. 20363781 10.1128/AEM.02612-09 PMC2876438

[66] Monk IR, Casey PG, Cronin M, Gahan CG, Hill C (2008) Development of multiple strain competitive index assays for *Listeria monocytogenes* using pIMC; a new site-specific integrative vector. BMC Microbiol 8: 96. 18554399 10.1186/1471-2180-8-96 PMC2440758

[67] Czuprynski CJ, Faith NG, Steinberg H, Neudeck B (2003) Sodium pentobarbital anesthesia transiently enhances the severity of infection following intragastric, but not intravenous, inoculation of *Listeria monocytogenes* in mice. Microb Pathog 35: 81–86. 12901847 10.1016/s0882-4010(03)00097-4

