# Supplementary figures and images for: InlA Promotes Dissemination of Listeria monocytogenes to the Mesenteric Lymph Nodes during Food Borne Infection of Mice
Source: PLoS Pathog. 2012 Nov 15;8(11):e1003015. doi: 10.1371/journal.ppat.1003015 (PMC3499570; doi:10.1371/journal.ppat.1003015)

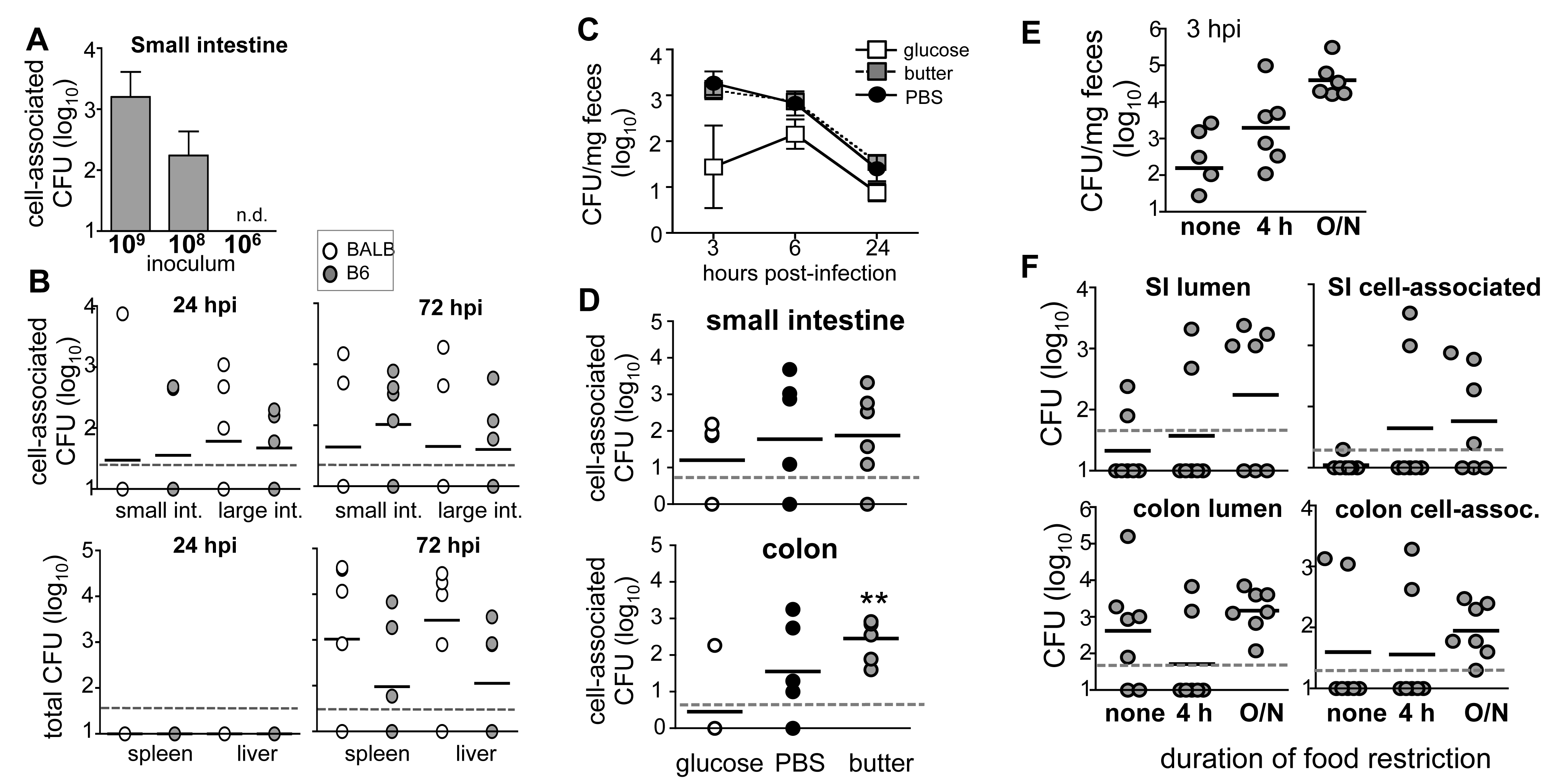

Supplement: Figure S1 — Optimization of the natural feeding model of L. monocytogenes infection. (A) Total cell-associated CFU in the small intestines of female B6 mice (n = 4) 24 h after ingestion of bread saturated with indicated dose of Lm InlAm. (B) BALB (white circles) and B6 (grey circles) mice (n = 4) were fed 3×10^8^ Lm InlAm and the cell-associated (intestines) or total CFU (spleen and liver) was determined 24 and 72 hpi. Bars indicate mean values for each group. (C, D) Female B6 mice (n = 6) were fed 3×10^8^ Lm InlAm suspended in either glucose, PBS, or melted butter at noon. Mean values +/− SD for Listeria shed in the feces (C) and the total cell-associated Listeria in the small intestine or colon 24 hpi (D) are shown. Asterisks indicate mean value significantly different from the mean for the glucose group, as assessed by unpaired t test. (E, F) Female BALB mice (n = 7) were denied food for 0 (none), 4 or 16 (O/N) hours and then fed bread pieces saturated with 3–5×10^8^ Lm InlAm suspended in butter. The total Listeria CFU present in the feces 3 hpi (E) and both the luminal and cell-associated L. monocytogenes in the small intestines and colon (F) was determined 24 hpi. The limit of detection for each organ is indicated by a dashed line. (TIF) [file ppat.1003015.s001.tif]

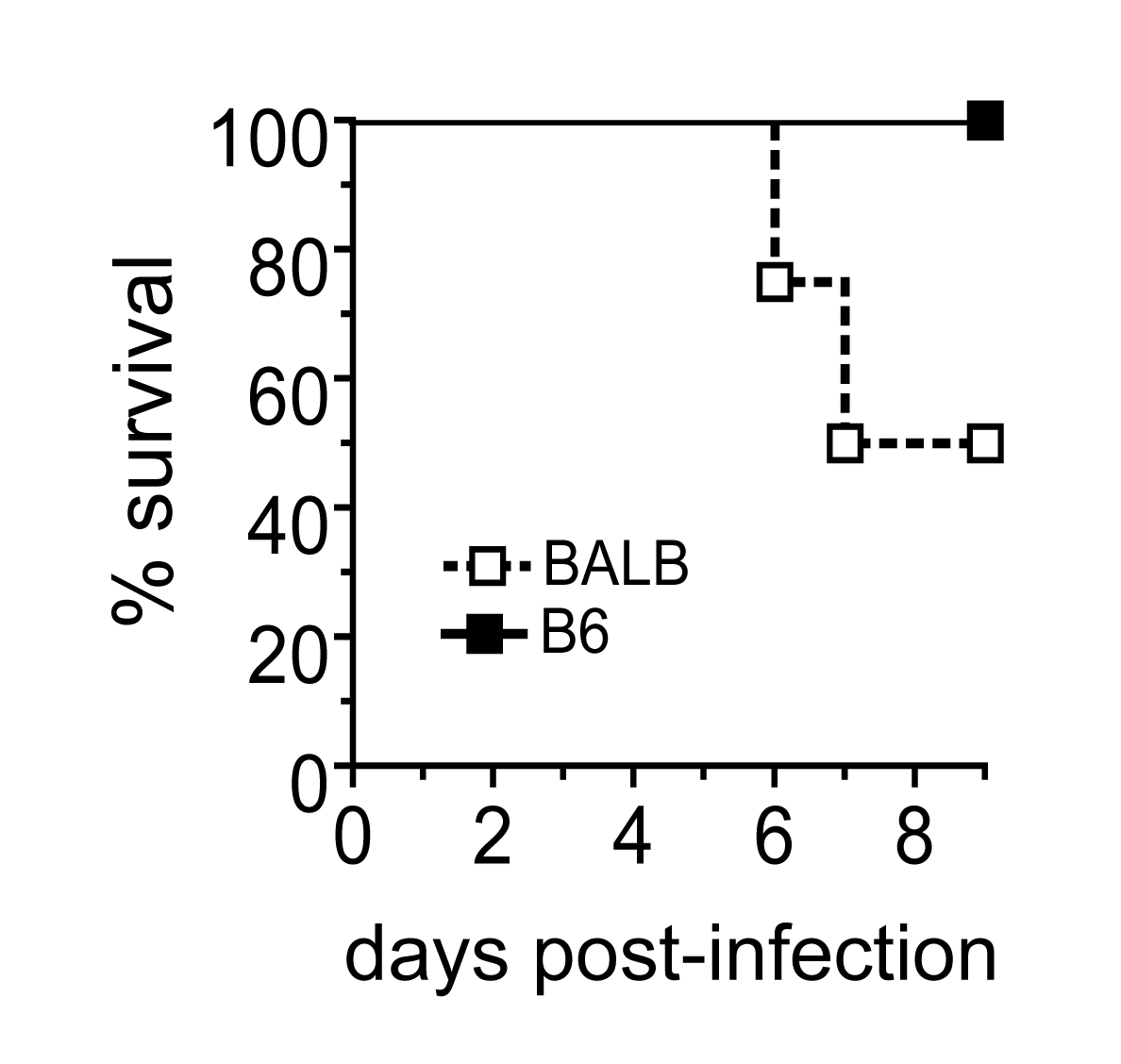

Supplement: Figure S2 — The LD50 for foodborne transmission in BALB mice is approximately 5×10^9^ CFU. Female BALB and B6 mice (n = 7) were fed 5×10^9^ Lm InlAm at night and survival was monitored over time. (TIF) [file ppat.1003015.s002.tif]

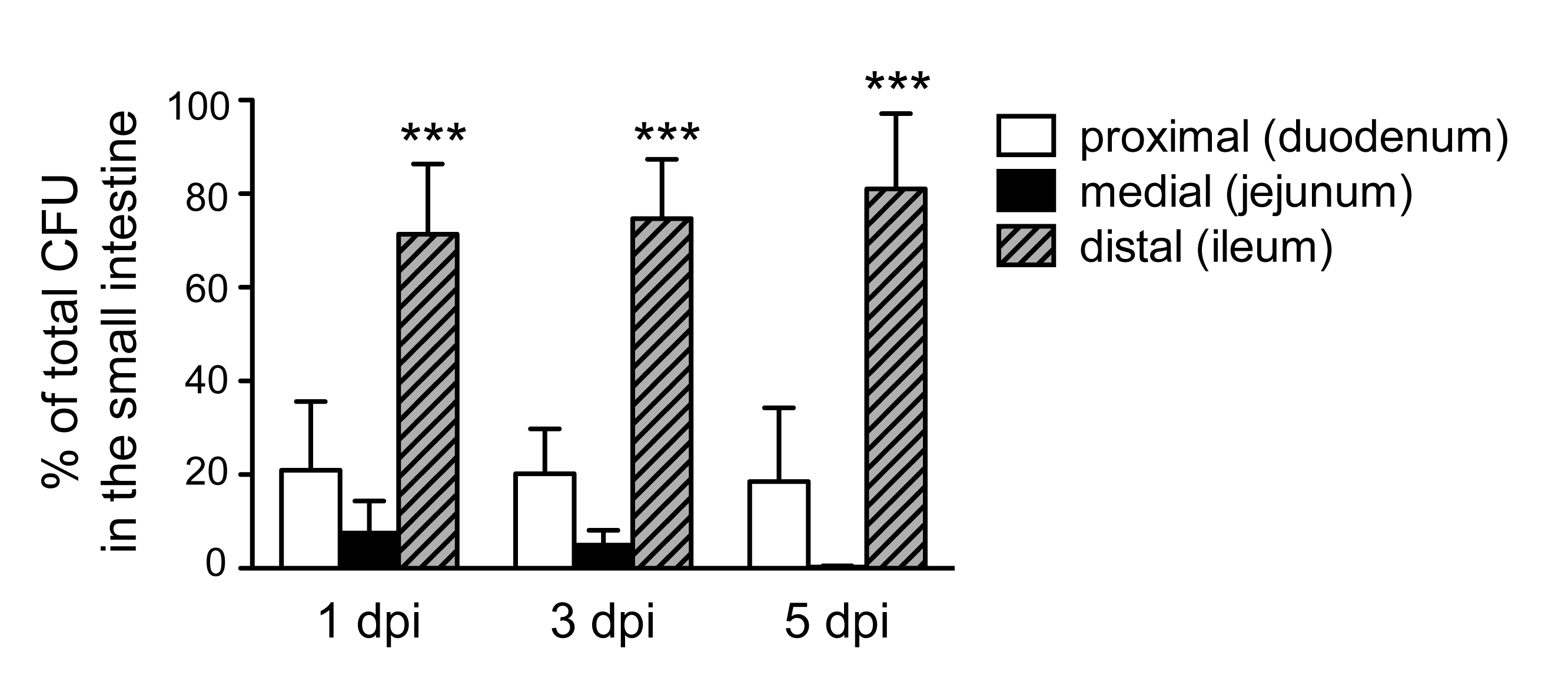

Supplement: Figure S3 — Food borne transmission of L. monocytogenes results in colonization of primarily the distal third of the small intestine. Female BALB mice (n = 4) were fed 1×10^9^ CFU of Lm InlAm and the total cell-associated CFU in the small intestine was determined over time. Each small intestine was cut into equal thirds approximating the duodenum (proximal), jejunum (medial), and ileum (distal) prior to flushing, homogenization, dilution and plating on BHI/L+G agar. Two-way ANOVA indicated a significant difference in bacterial colonization that varied with the section of the small intestine. (TIF) [file ppat.1003015.s003.tif]

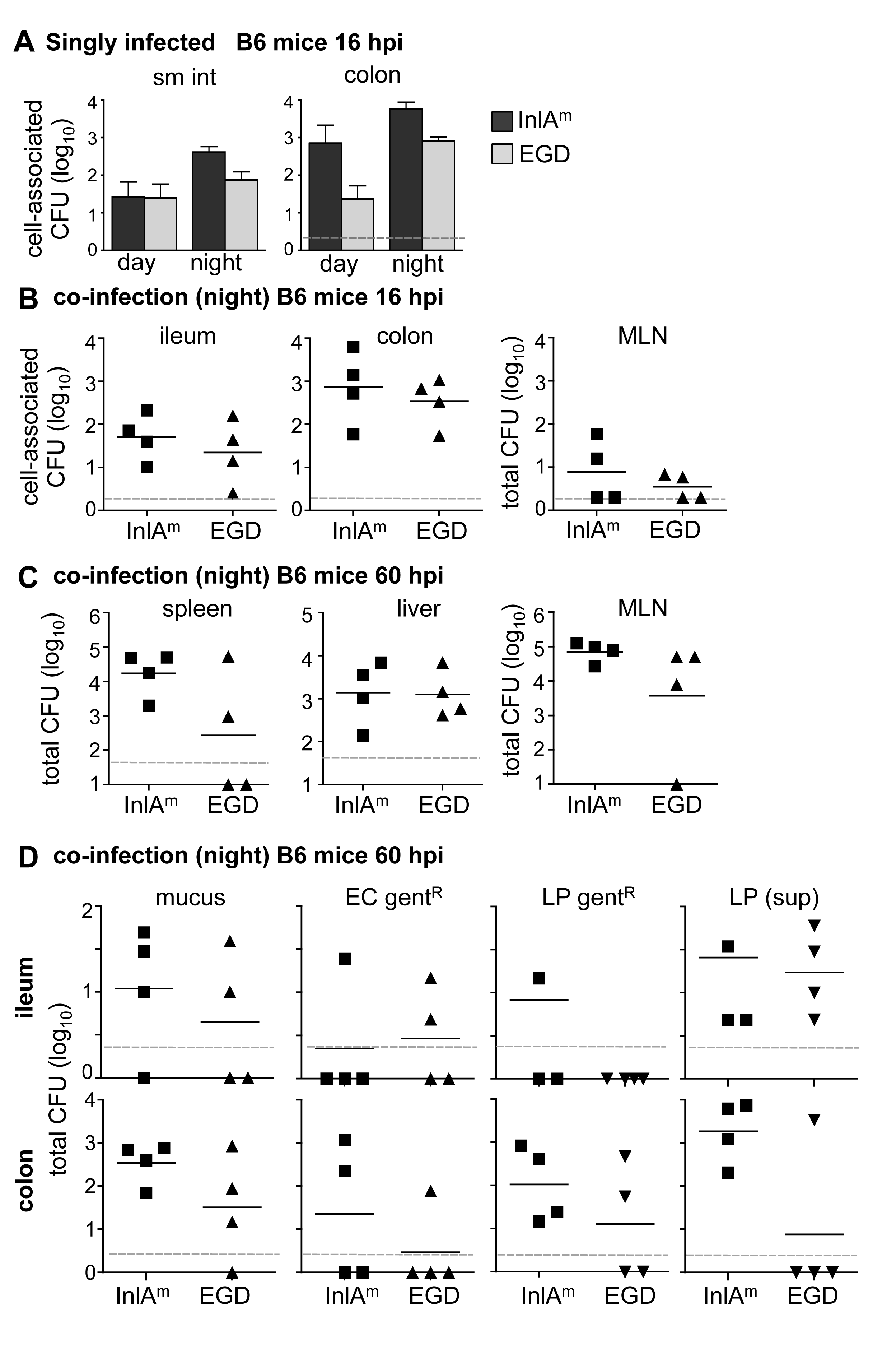

Supplement: Figure S4 — InlAm enhances systemic spread of L. monocytogenes in B6 mice. (A) Mean values +/−SD for total cell-associated Listeria in groups of female B6 mice infected either at noon (day) or 9:30 PM (night) with 4–5×10^8^ CFU of either Lm InlAm or Lm EGDe are shown. (B, C, D) Female B6 mice were co-infected with a total of 8–9×10^8^ CFU of Lm InlAm and Lm EGDe mixed in a 1∶1 ratio and total number of each strain present in the tissues 16 or 60 hpi was determined. In panel (D), ileum and colon were fractionated and the total mucus-associated and intracellular (gentR) or extracellular (sup) CFU in both the epithelial cell (EC) and lamina propria (LP) fractions were determined 60 hpi. Bars indicate mean values for each sample group. Dashed lines indicate the limit of detection in each organ. (TIF) [file ppat.1003015.s004.tif]
